# By-products of insect farming as a novel sustainable biofertilizer for crops

**DOI:** 10.1038/s44264-026-00175-4

**Published:** 2026-07-30

**Authors:** Katherine Y. Barragán Fonseca, Daan Mertens, Pedro Beschoren da Costa, Joop J. A. van Loon, Marcel Dicke

**Affiliations:** 1 Laboratory of Entomology, Wageningen University & Research, Wageningen, the Netherlands; 2 Grupo en Conservación y Manejo de Vida Silvestre, Instituto de Ciencias Naturales, Universidad Nacional de Colombia, Bogotá, Colombia; 3 Institute for Biodiversity and Ecosystem Dynamics, University of Amsterdam, Amsterdam, the Netherlands

**Keywords:** Ecology, Ecology, Environmental sciences, Microbiology, Plant sciences

## Abstract

The development of sustainable high-yield farming practices is crucial to support a growing human population while providing long-term solutions for the environmental impact of intensified agriculture. Nutrient-rich bio-residuals generated through the industrial production of insects hold a high but underexplored potential as an alternative to less sustainable fertilizers. In a two-year field experiment, we show that mustard plants grown in insect-exuviae-amended soil perform as well or even better than plants grown in soil amended with reference organic fertilizers. Improved plant performance was driven by increased plant growth in terms of height and width, a larger number of flowers produced, more interactions with pollinators, and a larger seed production compared to untreated plants. A parallel greenhouse experiment revealed that native root-associated microbial communities in exuviae-amended soil were more species-rich, less variable, and were characterized by several well-known plant-growth-promoting rhizobacteria compared to those found in unamended soil or soil treated with reference fertilizer. Collectively, these findings demonstrate that valorizing insect-based bio-residuals can improve agricultural sustainability while simultaneously supporting a circular economy.

## Introduction

Enhancing crop productivity by using agricultural inputs has been crucial in increasing food production to sustain the growing human population^1^. However, the use of fertilizers has also contributed to environmental deterioration through soil degradation, water pollution, and reduced nutrient absorption in soils, thereby threatening ecosystem services which are essential to agriculture^2–4^. Therefore, developing effective agricultural practices that boost plant productivity, which simultaneously safeguard ecosystem services and are in accordance with the United Nations’ Sustainable Development Goals (SDGs) will be essential to achieve long-term food security and support societal development^5–7^.

Organic agricultural inputs derived from industrial residual streams are of major interest in the development of strategies aiming to reduce the environmental footprint of agricultural systems by enhancing the sustainability and circularity of crop production^8^. In this context, the rapidly growing production of insects for food and feed holds a high but underexplored potential^9–12^. While the produced insects provide a sustainable source of protein for both human and animal consumption, the residual insect exuviae (molted exoskeletons) are rich in the polysaccharide chitin^13^. When added to soil, chitin is degraded to plant-growth-promoting nutrients containing nitrogen and phosphorus. The availability of nutrients also impacts plant performance, as more resources become available for promoting the plant’s development (e.g. the production of flowers) or by allocating resources to enhance the plant’s resistance to pests and diseases, for example, by inducing plant defenses^14,15^. In addition, chitin and its derivative chitosan act as biostimulants by promoting the growth and activity of beneficial plant-growth-promoting rhizobacteria (PGPR) such as *Bacillus* and *Pseudomonas* species naturally occurring in the soil, further boosting crop productivity^16^.

The changes in soil properties and microbial communities, triggered by the addition of chitin to soil, can thus directly affect a plant’s phenotype and, consequently, indirectly affect the plant’s interactions with its associated insect community^17^. These interactions are of major importance in agricultural contexts, as interactions with antagonists such as herbivores can decimate yields while interactions with mutualists such as pollinators constitute ecosystem services that are essential for qualitative and quantitative aspects of production^18,19^. Phenotypic modifications in plants resulting from the use of insect exuviae as biofertilizer, i.e., acting as both fertilizer and biostimulant, have been demonstrated for several ecologically relevant plant traits, including those exploited by insect pollinators^20^. For example, flowering plants benefit from augmented nutrient availability by increasing the flower corolla size, the number of flowers produced, and the display size of inflorescences, thereby enhancing the plant’s attractiveness to pollinators^21,22^. Likewise, through changes in visual or chemical plant traits that pollinators use as cues, chitin-induced changes in soil nutrients and microbial communities can indirectly affect the behavior and abundance of pollinators and alter the diversity of the pollinator community visiting plants. These changes in the interactions between plants and their associated pollinator community can affect the reproductive success of plants, as was shown for black mustard plants growing in soil amended with black soldier fly exuviae^20,23^.

Currently, the effects of insect-exuviae-based biofertilizers on plant development, pollinator attraction, and plant reproduction relative to the effects of conventional organic fertilizers such as cow manure have been especially assessed for black soldier fly exuviae^23,24^. However, exuviae from different insect species currently being mass-produced for food or feed, such as mealworms and house crickets, differ in chemical composition^13^. This may affect the direct and indirect effects of exuviae-based biofertilizers on plants and determine their effectiveness as organic fertilizers. Because of the high potential of insect exuviae as a sustainable organic fertilizer, assessing the effects of different exuviae-based biofertilizers on plant growth and on plant-mediated interactions is of major importance in the context of developing novel sustainable fertilization practices.

Our study takes a comparative approach by investigating the effects of supplementing soil with exuviae of three insect species, namely house cricket (*Acheta domesticus* L.), black soldier fly (*Hermetia illucens* L.), or mealworm (*Tenebrio molitor* L.)) on black mustard (*Brassica nigra* (L.) Koch) plants. These effects are compared to the effects of supplementing soil with a conventional organic fertilizer (cow manure) or a non-insect chitin-based fertilizer (shrimp chitin). In two years of common-garden experiments and a greenhouse experiment, we investigated whether plants grown on differently treated soil varied in (1) plant traits related to growth, biomass, and flowering, (2) the associated pollinator community, (3) seed yield, and (4) the associated rhizosphere bacterial community. We show that the effects of insect-exuviae-based biofertilizers on plant growth, pollinator attraction, and seed production are similar if not stronger than reference organic or chitin-based fertilizers, underscoring the high potential of insect exuviae as a sustainable source of organic biofertilizer.

## Results

We assessed whether supplementing soil with insect exuviae from one of three insect species, namely house crickets, black soldier flies, or mealworms, influences plant growth, pollinator interactions, seed production, and rhizosphere bacteria, and compared the effects of exuviae-based soil supplements with those induced when supplementing soil with shrimp chitin or organic fertilizer (cow manure), or when leaving soil untreated.

### Plant development

Type of soil treatment significantly affected the plant traits measured in the open field experiment (Table 1, Supplementary Fig. 1–4, Supplementary Table 2). Plants grown in soil supplemented with insect exuviae or organic fertilizer generally grew larger and wider, flowered earlier, and produced more flowers than control plants grown in untreated soil. This was not the case for plants growing in soil supplemented with shrimp chitin. Importantly, the development of plants grown in exuviae-supplemented soil was generally similar or even enhanced compared to the development of plants grown in soil supplemented with organic fertilizer. The only exceptions were related to plants grown in soil supplemented with black soldier fly exuviae, where we observed a reduced plant width and fewer flowers early in the growing season compared to plants grown in soil supplemented with organic fertilizer (Supplementary Figs. 2, 3).

**Table 1:** Overview of the effect size of soil treatment, year of the field season, and their interaction on plant traits and pollinator interactions observed in the field experiments

		Treatment			Year			Treatment * Year		
Response variable	Time point	DF	*χ*^2^	p	DF	*χ*^2^	p	DF	*χ*^2^	p
Plant height	Early	5	43.72	**0.0001**	1	5.89	**0.0153**	5	1.84	0.8709
	Late	5	33.10	**0.0001**	1	2.84	0.0919	5	3.61	0.6065
Plant width	Early	5	52.60	**0.0001**	1	2.27	0.1315	5	10.99	0.0516
	Late	5	67.63	**0.0001**	1	0.86	0.3455	5	25.50	**0.0001**
Number of flowers	Early	5	89.96	**0.0001**	1	0.25	0.4943	5	4.39	0.4943
	Late	5	104.71	**0.0001**	1	0.01	0.9222	5	2.53	0.7719
Days until flowering	Early	5	10.35	0.0659	1	2.84	0.0922	5	25.09	**0.0001**
Total pollinators	Aggregated	5	33.83	**0.0001**	1	17.86	**0.0001**	5	5.48	0.3601
Pollinator visits	Aggregated	5	17.23	**0.0041**	1	0.82	0.3665	5	17.39	**0.0038**
Number of seeds	End	5	65.60	**0.0001**	1	0.82	0.3661	5	7.31	0.1987

Time point refers to whether the data was collected in early-season or late-season, was aggregated for two observation rounds, or was collected at the end of the season when plants were harvested. We applied a log-transformation to data on the number of flowers, the number of days until flowering, the number of flowers visited per tracked pollinator (presented here as pollinator visits), and the number of seeds produced. Bold *P* values represent statistically significant effects.

**Fig. 1:**
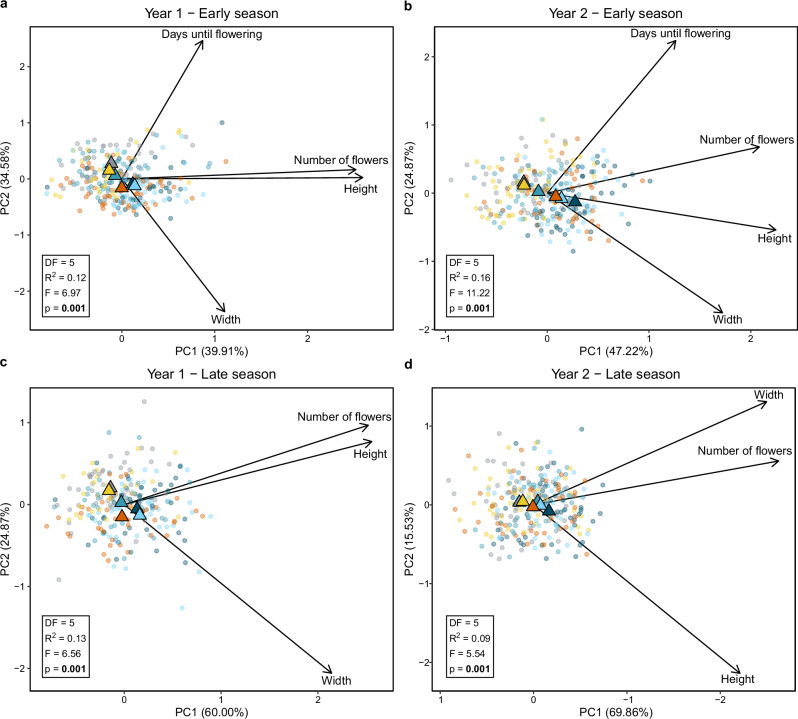
Ordination of the multivariate phenotype of Brassica nigra plants grown in soil with different treatments in two years of field experiments. Panels represent the principal component (PC) ordination of plant trait measurements taken **a** early-season in the first year of field experiments, **b** early-season in the second year of field experiments, **c** late-season in the first year of field experiments, and **d** late-season in the second year of field experiments. Plants were grown in soil supplemented with exuviae of one of three insect species (underlined treatments), namely house cricket (HC), black soldier fly (BSF), or mealworm (MW), were grown in soil amended with shrimp chitin (CHT) or organic fertilizer (OF), or were grown in unamended soil as a control treatment (C). Points represent the multivariate coordinates of individual plants, with triangles indicating the centroid of each treatment in multivariate space. Vectors indicate the direction of separation as structured by the respective phenotypic trait. Boxes in each panel represent the results of a PERMANOVA. The two principal components are annotated with the percentage of total variation they represent. Sample sizes are provided in Supplementary Table 1.

Congruent to the patterns observed when evaluating the phenotypic traits separately, we found that the multivariate representation of the measured plant traits was significantly affected by the type of soil treatment both early and later in the growing season (Fig. 1). Plants growing in soil supplemented with house cricket or mealworm exuviae or in soil supplemented with organic fertilizer developed a phenotype that significantly differed from that of control plants (Fig. 1, Supplementary Table 2). For plants growing in soil supplemented with black soldier fly exuviae, this was only the case later in the growing season of the second year of the experiments. Phenotypic traits of plants grown in soil supplemented with shrimp chitin were similar to those of the control plants in both years (Supplementary Table 2).

### Interactions with pollinators

**Fig. 2:**
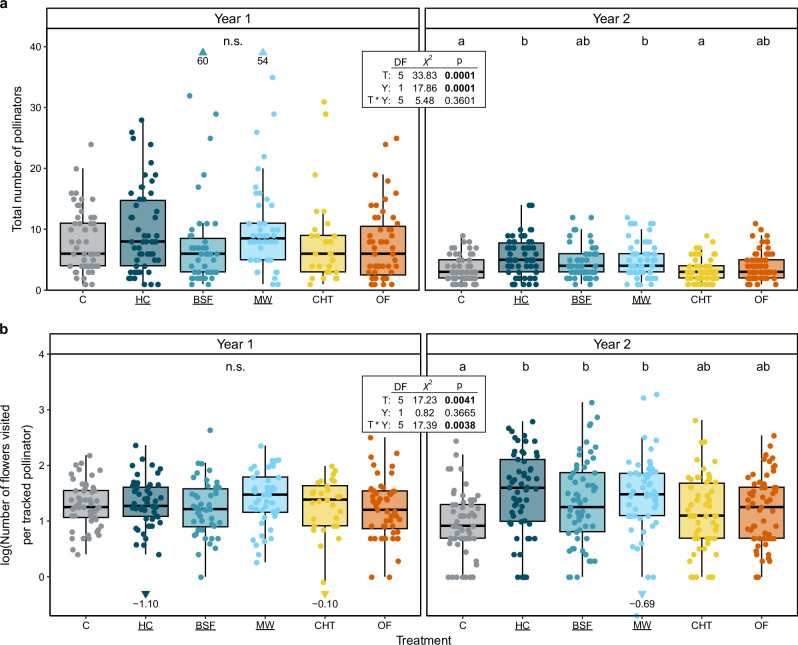
Pollinator interactions observed for Brassica nigra plants grown in soil with different treatments in two years of field experiments. Interactions are expressed by **a** the total number of pollinators visiting individual plants and **b** the number of flowers visited per tracked pollinator. Plants were grown in soil supplemented with exuviae of one of three insect species (underlined treatments), namely house cricket (HC), black soldier fly (BSF) or mealworm (MW). These are compared to observations for plants grown in soil supplemented with either shrimp chitin (CHT), organic fertilizer (OF), or control plants (C) grown in unamended soil. Boxplots represent the median and interquartile range (IQR); whiskers represent 1.5 × IQR. Points represent individual observations, with triangles annotated with their respective values indicating observations that lie outside of the figure range. Results of a Generalized Least Square model are presented in the boxes, where T is the main factor Treatment; Y is the main factor Year, and T*Y is the interaction between Treatment and Year. Lower case letters above box plots indicate significant pairwise differences at p < 0.05 if no letters are shared, whereas n.s. indicates that no significant pair-wise differences were found. Sample sizes are provided in Supplementary Table 1.

The type of soil treatment significantly explained variation in both the total number of pollinators visiting individual plants, as well as the number of flowers visited per tracked pollinator (Fig. 2, Supplementary Table 3). In the second year of field experiments, plants grown in soil supplemented with house cricket or mealworm exuviae interacted with more pollinators than control plants. In addition, the pollinators interacting with plants grown in soil supplemented with any of the three types of insect exuviae also visited a greater number of flowers per plant. While this effect seems to be present in year 1 as well, post-hoc analyses did not reveal significant contrasts among different soil treatments in that year. Importantly, plants grown in soil with insect-exuviae-based treatments were visited by as many pollinators as plants growing in soil supplemented with organic fertilizer, and tracked pollinators visited similar numbers of flowers per plant (Fig. 2). The type of soil treatment did not affect the composition of the pollinator community visiting individual plants (Supplementary Fig. 5).

### Plant- and pollinator-mediated effects on seed production

**Fig. 3:**
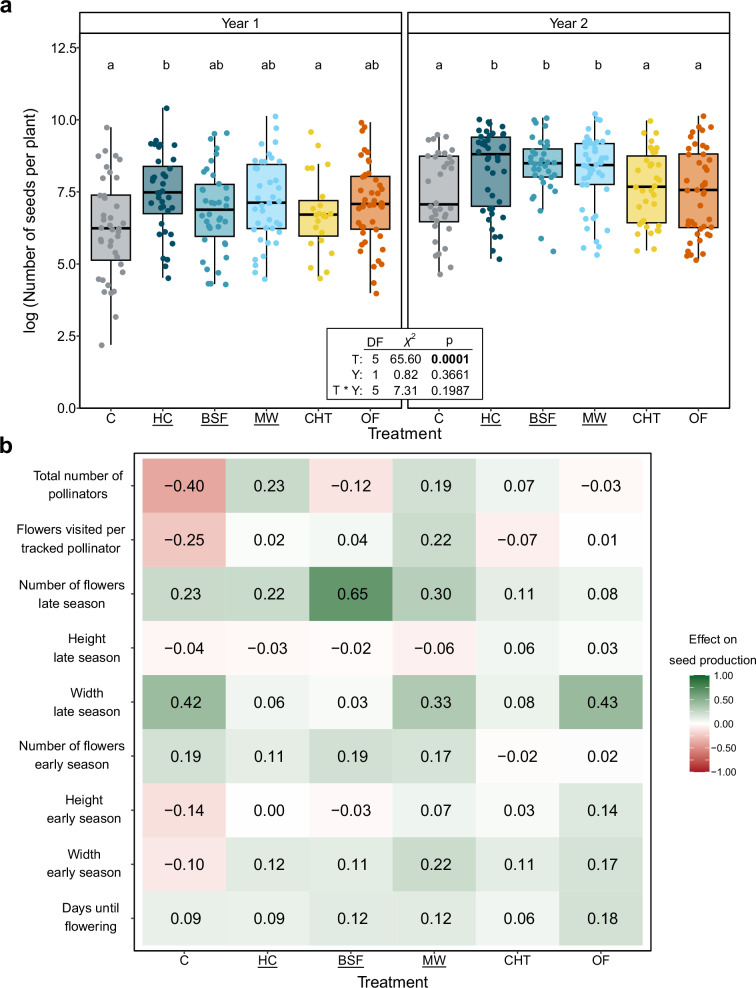
Overview of the estimated effects on seed production of *Brassica nigra* plants grown in soil with different treatments in the field experiment. Variation in seed production among individual plants can be **a** directly related to the soil treatment, and **b** interpreted as the result of cascading direct and indirect causal effects. Plants were grown in soil supplemented with exuviae of one of three insect species (underlined treatments), namely house cricket (HC), black soldier fly (BSF) or mealworm (MW), in soil supplemented with either shrimp chitin (CHT) or organic fertilizer (OF), or were grown in untreated soil as a control treatment (C). The box plots in panel a represent the median and interquartile range (IQR); whiskers represent 1.5 × IQR. Points represent individual observations. Results of a Generalized Least Square model are presented in the box, where T is the main factor Treatment; Y is the main factor Year, and T*Y is the interaction between Treatment and Year. Lower case letters above box plots indicate significant pairwise differences at *p* < 0.05 if no letters are shared, whereas n.s. indicates that no significant pair-wise differences were found. The heat map in panel b is color-coded to represent the total causal effect of variables included in the structural equation model on log-transformed seed production (Supplementary Fig. 6). Values can be interpreted as the relative effect a unit increase in the variable has on the log-transformed number of seeds produced by a plant. Sample sizes are provided in Supplementary Table 1.

The soil treatments significantly explain variation in the number of seeds produced by the plants (Fig. 3a). Plants growing in soil supplemented with house cricket exuviae produced more seeds than control plants in year 1. In year 2 plants growing in soil supplemented with any of the three types of insect exuviae produced significantly more seeds than either control plants, plants growing in soil supplemented with shrimp chitin, or plants growing in soil supplemented with organic fertilizer. Our hypothesis that early-season plant growth (i.e., height and width), late-season plant growth, plant development, interactions with the pollinator community, and finally seed production are causally linked, was supported by the data from each soil treatment (Supplementary Fig. 6, Supplementary Table 4). Variables in the path model generally had a positive causal effect on seed production, with the strongest effects on seed production associated with the height and width of plants late in the season and the two measures associated with the pollinator community (Fig. 3b, Supplementary Table 5). However, the sign of causal effects and the identity of the variables with the strongest causal relation with seed production varied among treatments. In general, we observed more negative causal effects on seed production for control plants compared to the other treatments, showing for example that a higher investment in plant height and width early in the growing season had a negative causal effect on seed production (Fig. 3b). We hypothesize that the counter-intuitive negative relationships observed between the variables in the causal model and the number of seeds produced by plants may result from unmeasured changes in the herbivore community. For example, an expected positive causal effect of increased interactions with pollinators on the number of seeds produced may be outweighed by a simultaneous and treatment-dependent increase in flower and seed predation by herbivores. In the insect-exuviae treatments, seed production was especially associated with the number of flowers produced and pollinator community metrics. In contrast, seed production of plants in the organic fertilizer treatment was more closely related to plant height and width. Finally, seed production of plants grown in soil amended with shrimp chitin was relatively unresponsive to variation in the development of plants or variation in interactions with the associated pollinator community.

### Rhizosphere microbial communities

**Fig. 4:**
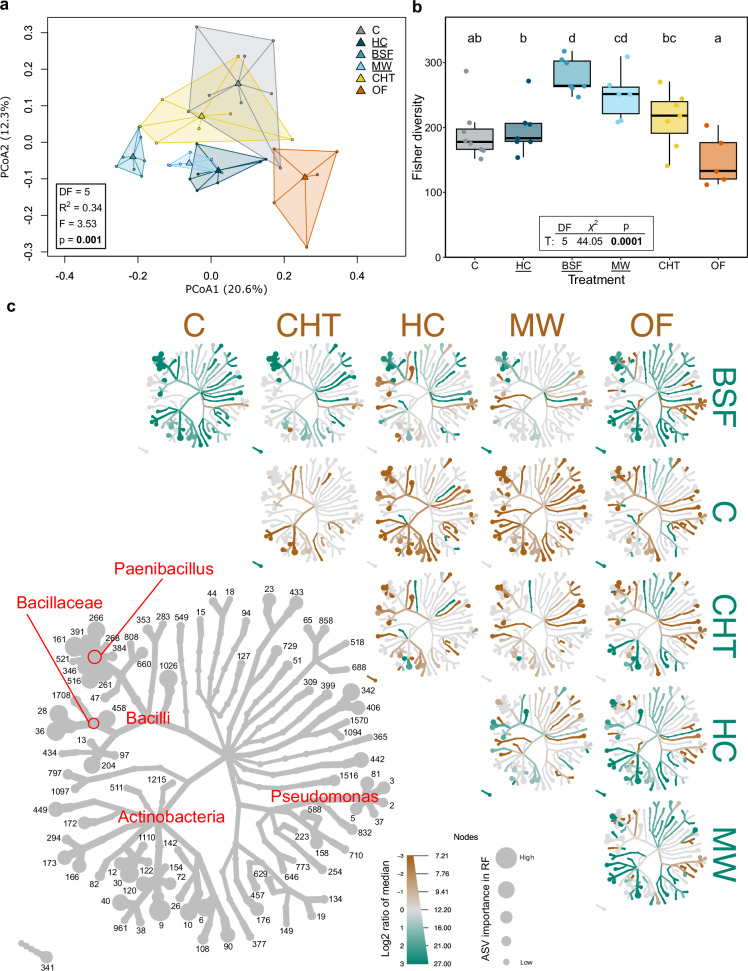
Overview of the analysis on microbial communities in the rhizosphere of plants grown in soil with different treatments in the greenhouse. **a** Principal coordinate analysis (PCoA) ordination based on Bray-Curtis distances between microbial communities. Different colors represent communities associated with *Brassica nigra* plants grown in soil supplemented with house cricket exuviae (HC), black soldier fly exuviae (BSF), mealworm exuviae (MW), shrimp chitin (CHT), organic fertilizer (OF), or plants grown in untreated soil (C). The three different exuviae-based soil amendments are underlined in the figure legend. Points represent the multivariate coordinates of communities associated with individual plants, with triangles indicating the centroid of each treatment in multivariate space. Shaded areas visualize the spread of samples in multivariate space. The two principal axes are annotated with the percentage of total variation they represent. The box shows the results of the PERMANOVA analysis **b** Fisher’s diversity index, indicating the effect of different soil treatments on the diversity of soil microbial communities. The boxes represent the quantile distribution (25th percentile, median, and 75th percentile) of Fisher’s diversity index, and whiskers span 1.5 × interquartile range. Points represent individual observations. Results of a GLS testing the effect of soil treatment (abbreviated as T) are presented in the box. Lower case letters above box plots indicate significant pairwise differences. **c** Matrix of heat trees comparing taxonomies and abundances of the 94 ASVs that are important in differentiating soil treatments according to the Boruta random forest model. Differently colored branches indicate significant pairwise differences in log_2_-transformed relative abundances across treatments, evaluated using a Wilcoxon Rank Sum test. The blue-green color represents higher taxa abundance in the treatment labeled in the rows, the brown color represents higher taxa abundance in the treatment labeled in the column, and the grey color indicates similarity in taxa abundance. The large grey tree to the left serves as a key to the legend. Each node represents a taxonomic group, with ASVs annotated with their respective number. Sample size is presented in Supplementary Table 1. A detailed version of panel c, including additional labeling from domain to ASV, is available in the online supplementary information.

Soil treatments significantly affected the structure of microbial communities in the plant rhizosphere (Fig. 4a), with each treatment resulting in distinctly different microbial communities (Supplementary Table 6). The microbial communities associated with the black soldier fly and organic fertilizer treatments exhibited the biggest pairwise difference. Interestingly, variation in the composition and structure of microbial communities among samples (*i.e*. beta dispersion) was significantly dependent on the soil treatment (F = 3.58, DF = 5, *p* = 0.01), with treatments using house cricket or mealworm exuviae as soil supplement showing significantly less variation when compared to the control treatment (Fig. 4a, Supplementary Table 7). The species richness of microbial communities weighted for species abundance, represented by Fisher’s and Shannon diversity indices, also significantly differed between treatments (Fig. 4b, Supplementary Fig. 7). Treatments with black soldier fly and mealworm exuviae had a higher microbial diversity compared to control plants, and all insect exuviae treatments had a more diverse microbial community compared to the organic fertilizer treatment.

The two different methods used to evaluate the effect of soil treatments on amplicon sequence variant (ASV) abundance (differential abundance testing and random forest analysis) presented interesting overlaps. By using differential abundance testing, we detected 62 differentially abundant ASVs, with samples separating into six distinct clusters based on a complete linkage algorithm (Supplementary Fig. 8). The most represented taxonomic groups are the class Actinobacteria (27 ASVs) and the order Paenibacillales (12 ASVs). In parallel, the random forest analysis identified 94 ASVs as important predictors of the soil treatment (Fig. 4c). Variation in the abundance of these 94 ASVs allowed the trained random forest model to correctly associate the observed community with the correct soil treatment with an accuracy of 95.1%. Of the 15 ASVs contributing most to the random forest model (*i.e*., of which variation was most characteristic in association with the different treatments), nine are in the class Bacilli and five in the class Actinobacteria. The microbial communities associated with plant roots grown in insect-exuviae-supplemented soil were distinguishable from other treatments by a group of 32 ASVs spanning clusters 1, 2 and 3, with Paenibacillales as the most common order. The abundance of *Paenibacillus* was highest in the soil treated with black soldier fly exuviae and lowest in the control treatment. House cricket and mealworm treatments are characterized by different Actinobacteria, whereas organic fertilizer treatment yielded the highest occurrence of *Pseudomonas*. The shrimp chitin treatment resulted in a community most similar to the control (Supplementary Table 6). Twenty-seven out of the 94 ASVs that were detected as important to differentiate treatments in the random forest analysis were also found to be differentially abundant in the alternative statistical approach (differential abundance testing). Here, Paenibacillales were the most represented group, with 7 out of the 27 ASVs identified by both statistical methods as important to differentiate the rhizosphere communities associated with treatments belonging to this order.

Furthermore, we identified 23 ASVs as random forest predictors of the number of flowers in a regression model (RMSE = 8.97, r² = 0.59, Supplementary Fig. 9). Firmicutes emerged as the most prevalent phylum included in the regression model, with 8 ASVs. However, ASVs in the class Bacilli and phylum Proteobacteria exhibited greater importance in the model. There were 13 ASVs identified as random forest predictors for both the number of flowers and treatment type (Supplementary Table 8). Notably, Burkholderiales played a more substantial role as a predictor of flower count compared to its predictive power for treatment type (Supplementary Fig. 9, Fig. 4c). We did not detect any random forest predictors for plant biomass.

Finally, we performed a network analysis of the 94 ASVs identified as important predictors of treatment by the random forest model (visualized in Supplementary Fig. 10). ASV_13 (class Bacilli) emerged as a keystone taxon and module hub, while ASV_521 (class Bacilli, genus *Paenibacillus*) was identified as a module connector (Supplementary Fig. 11a, b). This suggests that these ASVs play pivotal roles in shaping the overall community and network structure, with their abundance potentially influencing many other ASVs in the community. In total, nine modules were identified in the network, with module 1 being the most well-connected, featuring a higher number of edges and containing both the module hub ASV_13 and module connector ASV_521. Out of the nine modules, three modules correlated positively with the number of flowers (cluster 4, 5, and 6) and two modules correlated positively with plant biomass (clusters 6 and 8), while none of the modules had a negative correlation with either the number of flowers or plant biomass (Supplementary Fig. 11c). Module 6, which correlated positively with both the number of flowers and plant biomass, encompasses ASV_3 (*Pseudomonas*), ASV_26 (*Phycicoccus*), and ASV_30 (*Nocardioides*). These ASVs were also identified as important predictors of the number of flowers in the random forest regression. The distribution of these three highlighted ASVs, the module hub ASV_13, and the module connector ASV_521 (Supplementary Fig. 11d) indicates higher abundance in communities associated with soil supplemented with black soldier fly exuviae compared to soil of control and shrimp chitin treatments. ASV_3, a *Pseudomonas* sp., was also highly abundant in the organic fertilizer treatment.

## Discussion

**Fig. 5:**
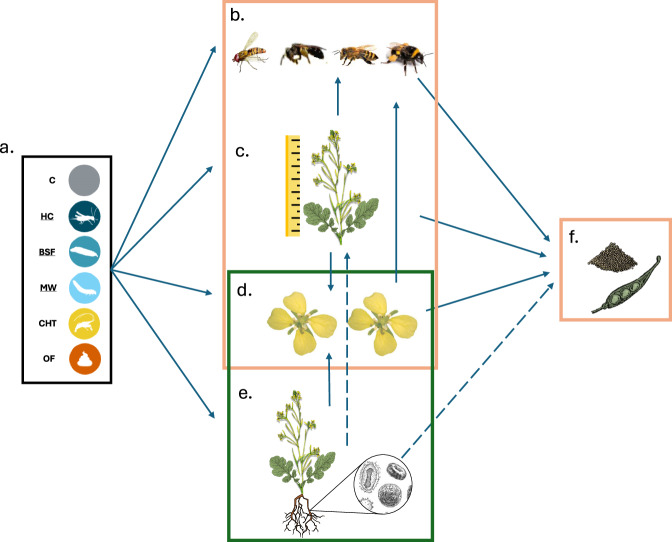
Summary of results obtained in the field and greenhouse experiments. The effects of different soil treatments **a** were tested in two years of field experiments (orange boxes) and a greenhouse experiment (green box). In the field experiments, soil treatments affected **b** the number of pollinators visiting individual plants and the number of flowers visited per tracked pollinator, affected **c** the plant phenotype in terms of height, width, and number of days until flowering started, and affected **d** the number of flowers plants produced. Structural equation models supported by data collected in the field experiments revealed a causal effect of the number of flowers and plant phenotype on interactions between plants and pollinators, and all variables affected **f** the number of seeds plants produced. In the greenhouse experiment, we found that soil treatments significantly affected **e** the composition, structure, and diversity of the root-associated microbial community, and the number of flowers produced by plants. Full arrows represent effects supported by data and statistical analysis, while dashed arrows represent relations between variables we hypothesize but did not test.

Our study shows that supplementing soil with insect exuviae promoted plant growth, enhanced pollinator attraction, and increased seed production in *B. nigra* plants compared to plants grown in untreated soil (Fig. 5). Importantly, these effects were as strong or even stronger than observed for plants growing in soil supplemented with reference organic or chitin-based fertilizers. In parallel, we show that microbial communities found in the rhizosphere of plants grown in insect-exuviae-treated soil were more species-rich, less variable, and distinct from those associated with plants grown in untreated or reference fertilizer soil treatments. Key micro-organisms differentiating the microbial community in exuviae-based soil treatments from those found in reference fertilizer and control treatments included members of classes and genera known to include plant-growth-promoting rhizobacteria (PGPR) such as Bacilli. The abundance of specific members of the microbial communities in exuviae-amended soil was also associated with flower production by plants (Fig. 5). Combined, our findings highlight the high potential of utilizing exuviae obtained as bio-residuals from industrial insect production to enhance plant growth and support sustainable crop production.

Our results reinforce findings from both lab and field experiments that insect-derived soil amendments can stimulate plant growth^24,25^. For example, *B. nigra* plants grown in soil supplemented with black soldier fly exuviae were reported to have a higher plant biomass, flower number, more interactions with pollinators, and an increased seed yield compared to plants grown in untreated soil^23^. In addition, we now extend this by showing that *B. nigra* plants grown in soil treated with insect exuviae not only outperform plants grown in untreated soil, but also frequently perform better than plants grown in soil supplemented with a reference organic or chitin-based fertilizer. Whether plants grown in exuviae-treated soil outperformed plants in the control or reference treatments was dependent on the type of exuviae used. Central to this context-dependency is that chitin from different organisms varies in the concentration of associated molecules such as proteins and fatty acids, leading to variation in nutrient availability in the soil as well as differential effects on the resident microbial community, as shown here^13^. As a result, the plant-growth-promoting effects of specific soil additions is influenced by the relation between the supplement-induced changes in plants or their rhizosphere microbiome, and how well these changes allow plants to match the environmental conditions they experience^26^. For example, the chitin derivative chitosan stimulates root hair callose deposition^27^. While this helps plants to cope with mild biotic stress, it also inhibits the growth and viability of root hairs, which is unfavorable when nutrients or water are limited. Importantly, as chitin cannot be used directly by plants, the plant-growth-promoting nutritional elements in insect-exuviae-based soil amendments only gradually become available through metabolic breakdown processes. This contrasts to traditional organic fertilizers such as cow manure, out of which free nutrients more readily leach, making plant-growth-promoting nutrients less consistently available to plants and with the additional risk of causing environmental eutrophication^28^.

Changes in the composition and structure of microbial communities associated with plant roots also play a pivotal role in the effectiveness of biofertilizers^29^. We found that supplementing soil with insect exuviae influenced the composition and structure of the microbial communities in the plant’s rhizosphere, and that supplementing soil with exuviae originating from different insect species led to the development of comparable but distinct microbial communities. Importantly, these microbial communities simultaneously increased in species diversity and reduced variation in community structure among samples (i.e. reduced beta dispersion) compared to the microbial communities observed in the control or reference fertilizer soil treatments. This higher microbial diversity and more consistent structure of communities are likely to lead to a more stable and consistent ecological functioning of the plant-associated microbiome ^30,31^. In addition, we found that several key microbial species differentiating exuviae-based soil treatments from the other soil treatments belonged to the class Bacilli, from which several members are well-known for their plant-growth-promoting effects ^32,33^. The importance of Bacilli-associated ASV’s in structuring the microbial communities, illustrated by our network analysis, emphasizes their role as key and interactive members of the rhizosphere community in exuviae-amended soil (Shi et al.^34^, Banerjee et al.^35^). While patterns in the effects of different soil treatments on microbial communities were obtained separately from results on plant performance in field experiments and direct extrapolation of results is not possible, we argue that changes in root-associated microbial communities are likely to be comparable under field conditions to those observed in the greenhouse experiment. In both experiments, plants were grown in soil collected from the experimental field. As the exuviae used in our experiments were heat-treated and thus effectively sterilized prior to their use as soil supplement, the distinct microbial communities and PGPR in the rhizosphere of differently treated plants result from the stimulation of native soil bacteria. The stimulation of native beneficial soil microorganisms has proven to be more advantageous than the inoculation of cultured microorganisms because these microbial inoculants often fail to establish in the new environment^36,37^.

Supplementing soil with insect exuviae also induced changes in the interactions between plants and their associated pollinator community. In the second year of the experiment, we found that plants growing in soil supplemented with mealworm or house cricket exuviae interacted with more pollinators compared to plants grown in untreated soil or in soil supplemented with reference chitin-based fertilizer. Moreover, pollinators interacting with plants grown in exuviae-treated soil visited more flowers compared to pollinators interacting with plants grown in untreated soil. The promotion of interactions between plants growing in exuviae-treated soil and the pollinator community can be explained by (a) the larger number of flowers produced by plants, and (b) changes in key plant traits such as flower reflectance and plant volatile emission, which are used as cues by pollinators^20,38^. We found no treatment-dependent changes in interaction strength between plants and specific pollinator groups, with the proportional composition of the pollinator community interacting with plants remaining similar among the treatments. Interestingly, while interactions with the pollinator community had a strong causal relation with seed production for plants grown in exuviae-supplemented soil, this was not the case for plants grown in soil supplemented with reference organic fertilizer. Here, variables related to plant biomass had a strong causal relation with seed production, while pollinator-related variables were less important.

We conclude that biofertilizers derived from the industrial production of a variety of insects hold a high potential as a sustainable alternative to traditional fertilizers. We show that, in addition to the known direct benefits of increased nutrient availability, exuviae-based soil supplements induce key changes in interactions with both the root-associated microbial community, as well as the flower-associated pollinator community. Both the stimulation of PGPR and the increased interactions with pollinators are likely key in boosting crop productivity and are recognized as key components of strategies aiming to reduce the environmental footprint of agricultural systems^39–41^. Importantly, the use of insect exuviae in our soil treatments reflects concentrations which can feasibly be applied in large-scale agricultural contexts. For example, we used 2 g of insect exuviae per kg of soil, while an equal concentration of nitrogen is only obtained when amending soil with 6.6 g of cow manure per kg of soil. As the large-scale production of insects as a sustainable source of animal protein is a rapidly growing industry^12,42^, valorizing by-products to strengthen a circular economy while simultaneously improving the sustainability of food production, thus holds great potential in the context of the development of sustainable agricultural strategies. Such strategies result from a local circular production system and therefore not only provide a sustainable crop fertilizer but also release crop production from the effects of volatile geopolitical disruptions of synthetic fertilizer production^43^.

## Methods

To assess whether supplementing soil with exuviae from three different insect species affects plant growth, plant development, the interactions with the pollinator community, the plant’s seed production, and the bacterial community associated with the plant rhizosphere, compared to supplementing soil with shrimp chitin, organic fertilizer, or leaving soil untreated, we set up two separate experiments. A first experiment was conducted in a common-garden set-up during two consecutive years in an experimental field (managed by Unifarm, and located in Wageningen, the Netherlands; 51°59'26.5“N, 5°39'50.5“E). This experiment aimed to assess the effects of the different types of soil treatments on plant development, interactions with the pollinator community, and plant reproduction under (semi-)natural conditions. The second experiment, conducted in a greenhouse, aimed to examine whether different types of soil treatments affected the plant-associated rhizosphere community and to link this variation in communities to changes in plant biomass and the number of flowers plants produced.

**Fig. 6:**
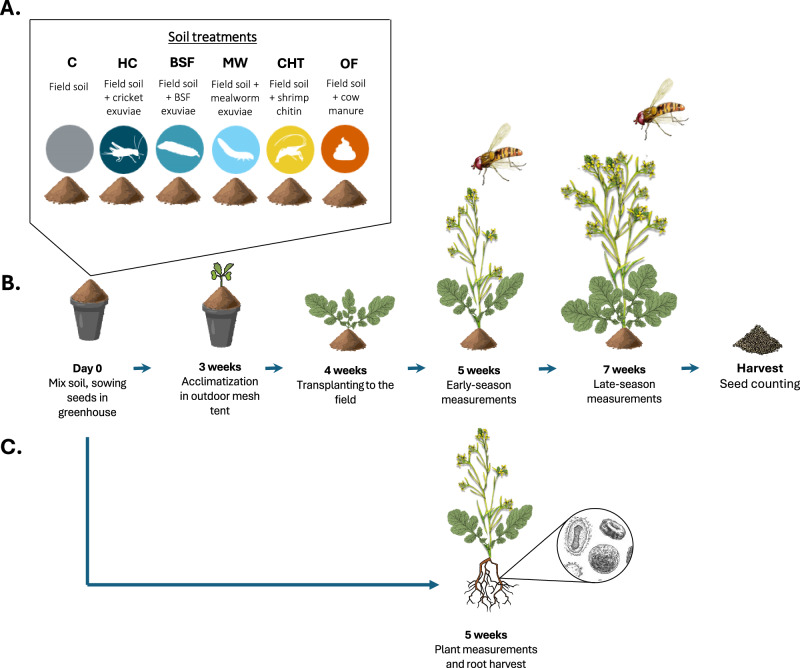
Schematic representation of the experimental design. **A** Soil collected from the experimental field was treated with house cricket exuviae (HC), black soldier fly exuviae (BSF), mealworm exuviae (MW), shrimp chitin (CHT), organic fertilizer (OF), or did not receive any supplements (C). Seeds of *Brassica nigra* were sown in treated soil and kept under greenhouse conditions. **B** When three weeks old, plants used in the field experiment were placed outside in mesh tents to allow acclimatization and were transplanted to the field at four weeks old. At five weeks old, we took early-season measurements including plant height and width, the number of days until flowering, and the number of flowers plants produced. In addition, we made observations on the pollinator community, including the total number of pollinators and the number of flowers visited per tracked pollinator. We repeated these measurements when plants were seven weeks old, i.e., late-season observations. At the end of the growing season, we harvested ripe siliques and counted seeds. **C** Plants used in the greenhouse experiment were kept until they were five weeks old. We then counted the number of flowers and harvested the full root system for analysis of the root-associated micro-community.

In both experiments, we used black mustard (*Brassica nigra* (L.) Koch, Brassicaceae) as a focal plant species. *Brassica nigra* is an annual plant native to Europe that interacts with a wide range of insects^44,45^. Seeds were obtained from the Centre for Genetic Resources (CGN, Wageningen, the Netherlands; accession number CGN06619) and were propagated by open pollination. Seeds were randomly assigned to one of six soil treatments. Soil treatments were created by collecting soil from the experimental field that was sieved (4 mm mesh size) to remove pebbles (Fig. 6). We then supplemented soil with insect exuviae by mixing exuviae of one of three insect species with the sieved soil: black soldier fly (*Hermetia illucens* L.; Diptera: Stratiomyidae; abbreviated as BSF), house cricket (*Acheta domesticus* L.; Orthoptera: Gryllidae; abbreviated as HC), or yellow mealworm (*Tenebrio molitor* L.; Coleoptera: Tenebrionidae; abbreviated as MW). Insect exuviae were provided by Bestico (Berkel en Rodenrijs, the Netherlands), Fair Insects (Dongen, the Netherlands), and Nijenkamp (Hellendoorn, the Netherlands), respectively. Exuviae were oven-dried at 60 °C for 24–48 h until a constant weight was reached. Exuviae were pulverized into a fine powder using a TissueLyser II stainless steel ball mill (QIAGEN, Germantown, Maryland, USA). To assess the effect of conventional fertilization on the plants, we used powdered cow manure (POKON, Spijkenisse, the Netherlands) as an organic fertilizer treatment (abbreviated as OF), and to assess the effect of a non-insect-based source of chitin, we used the powder of purified shrimp chitin (Sol-Actif, Orange, France) as chitin treatment (abbreviated as CHT). The control treatment (abbreviated as C) consisted of the sieved field soil without a supplement. For insect exuviae treatments, we used 2 g per kg of soil^23^. To have a nitrogen concentration equal to that of the treatments with insect exuviae, we used 6.6 g of cow manure per kg of soil in the organic fertilizer treatment (nitrogen content (g / kg): HC: 57.3, BSF: 72.8, MW: 92.3, and OF: 0.185). To have a chitin concentration equal to that of the treatments with insect exuviae, we used 0.2 g of shrimp chitin per kg of soil in the CHT treatment (chitin yield (%), (weight of chitin/weight of the powder) * 100: HC: 7, BSF: 8.1, MW: 9.88, and CHT: 99). Seeds were sown in 1 L pots placed individually in saucers in a greenhouse compartment (22 ± 1 °C, 50-70% RH, L16:D8) (Fig. 6). Natural daylight was supplemented with 400-Watt metal halide lamps (200 µmol m^−2^ s^−1^) when photosynthetically active radiation (PAR) dropped below 400 µmol m^−2^ s^−^^1^. Plants were watered twice per week by pouring water in the saucers until the topsoil became moist.

### Common-garden experiment

One week before planting in the field, the plants were acclimatized to natural weather conditions in an outdoor mesh tent, i.e., without climate control and excluding biotic interactions. Four-week-old plants were transplanted to the experimental field (Fig. 6, Supplementary Fig. 13). Prior to installation of the field experiments, the soil was tilled to homogenize conditions. The experimental plants were placed in a 32 m x 20 m field surrounded by an edge (0.5 m wide) of flowering *B. nigra* plants to standardize edge effects, and a mesh fence to exclude larger vertebrate herbivores. Experimental plants were planted at a distance of 1.5 m from each other on three different planting dates, each two weeks apart. The field had 400 plants in year 1 and 388 plants in year 2, with treatments being completely randomized among planting positions for each of the planting dates. The exact location of the experimental farm where the experiment was installed differed between the two years.

To investigate whether the type of soil amendment affected plant growth, we measured different plant traits as proxies for plant biomass in a non-destructive way. At two time points (5 and 7 weeks after seeds were sown, indicated as early season and late season, respectively) we recorded the width of the plant at its widest point (leaf tip to leaf tip). Between 7 and 9 days (early season; on average 5 weeks and 4 days after sowing) and 14–16 days (late season; on average 6 weeks and 4 days after sowing) after the first flower had opened, we measured plant height from the ground to the tip of the tallest inflorescence. In addition to these proxies for plant biomass, we recorded the number of days until the first flower opened and the total number of flowers on the sampling date as proxies for plant development rate.

To examine whether soil treatments influenced pollinator attraction to the plants or pollinator behavior while visiting flowers, we recorded pollinator visitation to the flowers of *B. nigra* plants in the six treatment groups. We recorded pollinator visitation at two time points: a first time within 7 – 9 days and a second time at 14–16 days after the first flower of the plant had fully opened. Each plant was monitored for a 10-min period per time point. Pollinator activity was recorded using a handheld computer (Psion Workabout Pro™ 3, London, UK) programmed with the Observer XT software (version 10, Noldus Information Technology, Wageningen, the Netherlands). When a pollinator made contact with a flower, its identity was recorded as one of the following: honeybee (*Apis mellifera* L.; Hymenoptera: Apidae), bumblebee (*Bombus* spp.; Hymenoptera: Apidae), syrphid fly (Diptera: Syrphidae), solitary bee (all Apidae excluding *A. mellifera* and *Bombus* spp.), or other flies (other Diptera than Syrphidae). In addition to the visitor’s identity, we recorded the number of flowers it visited. If other pollinators visited a plant during the observation of a particular pollinator, only the identity of those other pollinators was recorded. If the same pollinator individual returned to the observed plant after having visited a different plant, we recorded this as a new visit.

To assess whether soil treatments affected plant reproduction, we compared the seed production of plants in the six different treatment groups. Siliques were harvested when they were fully dry and brown but had not yet dehisced. Immature siliques and flowers were left on the plants and regularly checked for ripeness. Siliques and seeds were harvested in paper bags and kept in a dry storage room (20 °C), after which the seeds were manually extracted from siliques and cleaned. We counted the total number of seeds using an electronic seed counting machine (Contador, Pfeuffer GmbH, Germany).

### Greenhouse experiment

Rhizosphere samples were collected from eight plants per treatment when plants were five weeks old as described in ref. ^46^ (Fig. 6). The roots were manually separated from the loose soil by kneading and shaking. The entire root systems, along with a soil layer approximately 1 mm thick attached to their surface, were collected in 50 mL tubes containing 25 mL of sterile phosphate buffer (6.33 g NaH_2_PO_4_·H_2_O, 10.96 g Na_2_HPO_4_·2H_2_O, and 200 µL Silwet L-77 per L). The tubes were then vortexed for 15 s at maximum speed, after which the roots were removed, and the resulting suspensions were centrifuged for 15 min at 1800 *g*. The supernatants were discarded, and the pellets were stored at −25 °C until further processing for DNA isolation. DNA was extracted from 50 mg of each rhizosphere sample using the DNeasy PowerSoil Pro Kit (QIAGEN, Venlo, the Netherlands), and the quantity and quality of the DNA were assessed using the DeNovix DS-11 Fluorometer and dsDNA Broad Range assay (DeNovix, Wilmington, Delaware, USA). Rhizosphere samples that did not yield sufficient DNA for analysis were excluded. Five to eight samples per treatment were processed for DNA extraction and subsequent quantification and sequencing of the 16S rRNA genes. To explore the relation between variation in rhizosphere communities and the effect on plant development, we counted the number of flowers at the time of soil sample collection. As collecting the rhizosphere samples is destructive for the plants, we did not further monitor plant development or seed production in the greenhouse.

We obtained 3.76 M pair-ended 250 bp sequences from a total of 46 samples collected in the greenhouse experiment. After filtering, denoising, and removing plastid and chloroplast ASVs, ASVs occurring less than 8 times, and samples with less than 6000 sequences, we retained 40 samples for subsequent analyses (number of samples per treatment: C = 8; HC = 7; BSF = 7; MW = 6; CHT = 7; OF = 5), with a library size of 61,070 ± 9,661 reads for each sample (Supplementary Fig. 12). The 16S rRNA gene library preparation and sequencing were carried out by Genome Quebéc (Canada) on the Illumina MiSeq platform (PE 250 bp). The V4 region was targeted using the primers 515 f (5’-GTGCCAGCMGCCGCGGTAA) and 806r (5’-GGACTACHVGGGTWTCTAAT). To block plant DNA amplification, PNA clamps were included. We used Trimmomatic v 0.4 to remove low-quality sequence ends, and Cutadapt for primer sequence removal. Dada2 was used for sequence merging and denoising, and classify-sklearn was used for taxonomy assignment. We used Qiime2 2021.2 for all these processes. The SILVA 138 SSU database was used to train the classifier with standard configurations using Rescript. Raw sequences are available on the SRA database from NCBI with accession number SUB13848333.

### Statistical analysis

For each response variable, we first selected the best-fitting model from a set of candidate models based on the Akaike information criterion^47^. All candidate models included the type of soil amendment, the year in which the field experiment was conducted (year 1 or year 2), and their interaction as explanatory variables, and included the block in which plants were planted as a random intercept. Candidate models differed in terms of their probability distributions. We compared models with Gaussian and gamma distributions, and where relevant, Poisson and negative binomial distributions fitted using (generalized) linear mixed models ((G)LMMs). LMMs were further extended to account for potential heterogeneity of variance across the different treatments, the two years, or both factors by fitting generalized least-squares models (GLS)^48^. We used diagnostic plots to verify that model assumptions were met. In the analyses of the number of days until flowering started, the number of flowers produced, and the total number of seeds produced, none of the candidate models met the model assumptions. We therefore log-transformed these response variables and repeated the model selection procedure. We then estimated the significance of fixed factors using type II Wald χ^2^-tests. Finally, we evaluated pairwise post-hoc comparisons by Tukey’s honest significant difference (HSD) test, with contrasts considered significantly different at p ≤ 0.05. These analyses were done using the *emmeans*^49^, *glmmTMB*^50^, *lme4*^51^, *car*^52^, and *nlme*^53^ packages in *R* v.4.0.0^54^, and *R* Studio v. 1.4.1106^55^.

To summarize and compare the variation in the development of plant traits among the different treatments, we used a multivariate ordination (principal component analysis, PCA). To avoid visualizing the variation in plant development between the two years and eliminate pseudo-replication introduced by repeated measurements on the same plant, we analyzed each year-by-timepoint (indicated as early and late-season observations) combination separately. Ordinations were performed using Euclidean distance matrices calculated for scaled plant traits (mean = 0; SD = 1), using the *vegan* package in R^56^. We then estimated the overall dissimilarity in plant traits explained by the treatments by performing a permutational analysis of variance (PERMANOVA), including the dependency of observations introduced by the planting blocks in our permutational design^57^. Statistical significance was assessed via 999 permutations. Post-hoc comparisons between plant species were made by running a separate PERMANOVA analysis for each comparison and limiting the proportion of Type I errors by false discovery rate control^58^.

Initial data exploration revealed that interactions with several of the functional pollinator groups in our classification were relatively uncommon for *B. nigra*, either in general or at one of the two observation rounds. Most interactions with flowers were dominated by syrphid flies and honeybees, while solitary bees and bumblebees were observed infrequently. Similarly, representatives of the group categorized as “other flies” were rarely encountered, rendering temporal separation of observations in the two monitoring rounds (7 to 9 days and 14 to 16 days after flowering started) impractical for these groups. In addition, patterns were highly consistent across the two rounds of observations (Supplementary Fig. 5). Hence, to better match subsequent analyses, we chose to continue our analysis on the total number of pollinators and the average number of flowers visited per tracked pollinator (total number of flowers visited / total number of tracked pollinators), summed for both rounds of observations and independent of the functional group the pollinator belonged to. Models on interactions with the pollinator community (number of visitors and number of flowers visited per tracked pollinator) included the type of soil amendment, the year in which the field experiment was conducted (year 1 or year 2), and their interaction as explanatory variables, and included the block in which plants were planted as a random intercept. We followed the same model selection and estimation procedure outlined in the analysis of plant traits and seed production. The total number of pollinators was best modeled using a GLMM with a Gamma probability distribution and log link function. To meet model assumptions, we log-transformed the average number of flowers visited per tracked pollinator and fitted a GLS model with variance functions accounting for the heterogeneity of residuals across all years and treatment levels. The bipartite interaction network was constructed using the *bipartite* package in *R*^59^.

To disentangle the direct and indirect causal effects of plant height and width and pollinator activity on seed production of plants, we formulated a path model^60^ that related plant size (*i.e*. height and width) early in the season, plant size late in the season, the number of flowers produced by plants early and late in the season, the number of days until flowering started, and the two measures describing interactions with the pollinator community, to seed production of individual plants. The path model was constructed by specifying Generalized Least Square (GLS) models, including the identity of blocks as a random effect, and accounting for the heterogeneity of residuals by using variance functions, resulting in a better model fit based on AIC. The path model was then evaluated by Fisher’s C global goodness of fit statistic with the associated *p*-value, where *p* > 0.05 indicates that the data are sufficiently well represented by the path model 60, 61. We then compared the dependency of direct and indirect effects of variables included in the path model on the soil treatment by fitting the causal model to the subset of the data collected for each treatment. This analysis was performed using the *piecewiseSEM* package in *R*^61^.

To normalize library sizes for beta diversity analysis, we used the *MetagenomeSeq* package’s Cumulative Sum Scaling method^62^, and for analysis of Fisher’s and Shannon diversity, samples were rarefied to 47,000 reads using the *phyloseq* package^63^. The betadisper function of the *vegan* package was used to calculate a PCoA ordination of Bray-Curtis distances between microbial communities. The significance of community differences caused by treatment effects was assessed using the functions adonis2 from *vegan* and Adonis.pair from the *EcolUtils*^64^ package using 999 permutations and *p*-value correction via false discovery rate^58^. The Fisher and Shannon diversity indices were computed using the diversity function of the *microbiome* package^65^, and treatment effect significance was evaluated using the functions “aov” from the *stats* package^54^ and LSD.test function from the *agricolae* package^66^. The *DESeq2* package was used for differential abundance analysis^67^. Random forest feature selection for a regression task was performed using the *Boruta* package^68^. This was used to select features (ASVs) that could clearly outperform shadow features (a data-derived background) in predicting plant phenotype (number of flowers and biomass) or soil amendment in groups of random decision trees, which take ASV abundance as an input. This input was based on rarefied data and model performance was evaluated using a 5-fold cross-validation that was repeated 200 times with the *caret* package^69^. Co-occurrence networks of features selected by random forest were constructed with the *bdgraph* package based on 4.5 million iterations^70^. The network was filtered to retain only the 5% most probable edges. Network components were evaluated based on module connectivity and keystone definitions (Banerjee et al.^71^, Olesen et al.^71^). Correlations of plant metadata with network modules were calculated with the *WGCNA* package^72^.

## Supplementary information

Supplementary information

## Data Availability

All data supporting the results presented in this work are available from the following Zenodo digital repositories: field and greenhouse data 10.5281/zenodo.20942448 and sequencing data 10.5281/zenodo.20942577.

## Code availability

All code supporting the results presented in this work is available from the following Zenodo digital repository: 10.5281/zenodo.20942448.
