# Supplementary material for: By-products of insect farming as a novel sustainable biofertilizer for crops
Source: NPJ Sustain Agric. 2026 Jul 30;4(1):62. doi: 10.1038/s44264-026-00175-4 (PMC13423803; doi:10.1038/s44264-026-00175-4)
Supplement: Supplementary file 1 — Supplementary information [file 44264_2026_175_MOESM1_ESM.pdf]

Supplementary Information for

By-products of insect farming as novel sustainable biofertilizer for crops

Katherine Y. Barragán Fonseca[1,2]†, Daan Mertens[3]†, Pedro Beschoren da Costa[1], Joop J.A. van Loon[1] and Marcel Dicke[1]*

1 Laboratory of Entomology, Wageningen University & Research, Wageningen, the Netherlands

2 Grupo en Conservación y Manejo de Vida Silvestre, Instituto de Ciencias Naturales, Universidad Nacional de Colombia, Bogotá, Colombia

3 Institute for Biodiversity and Ecosystem Dynamics, University of Amsterdam, Amsterdam, the Netherlands

* Correspondence to: marcel.dicke@wur.nl

† These authors contributed equally to the work

This PDF file includes:

Supplementary Figures (Fig. S1-S13)

Supplementary Tables (Tables S1-S8)

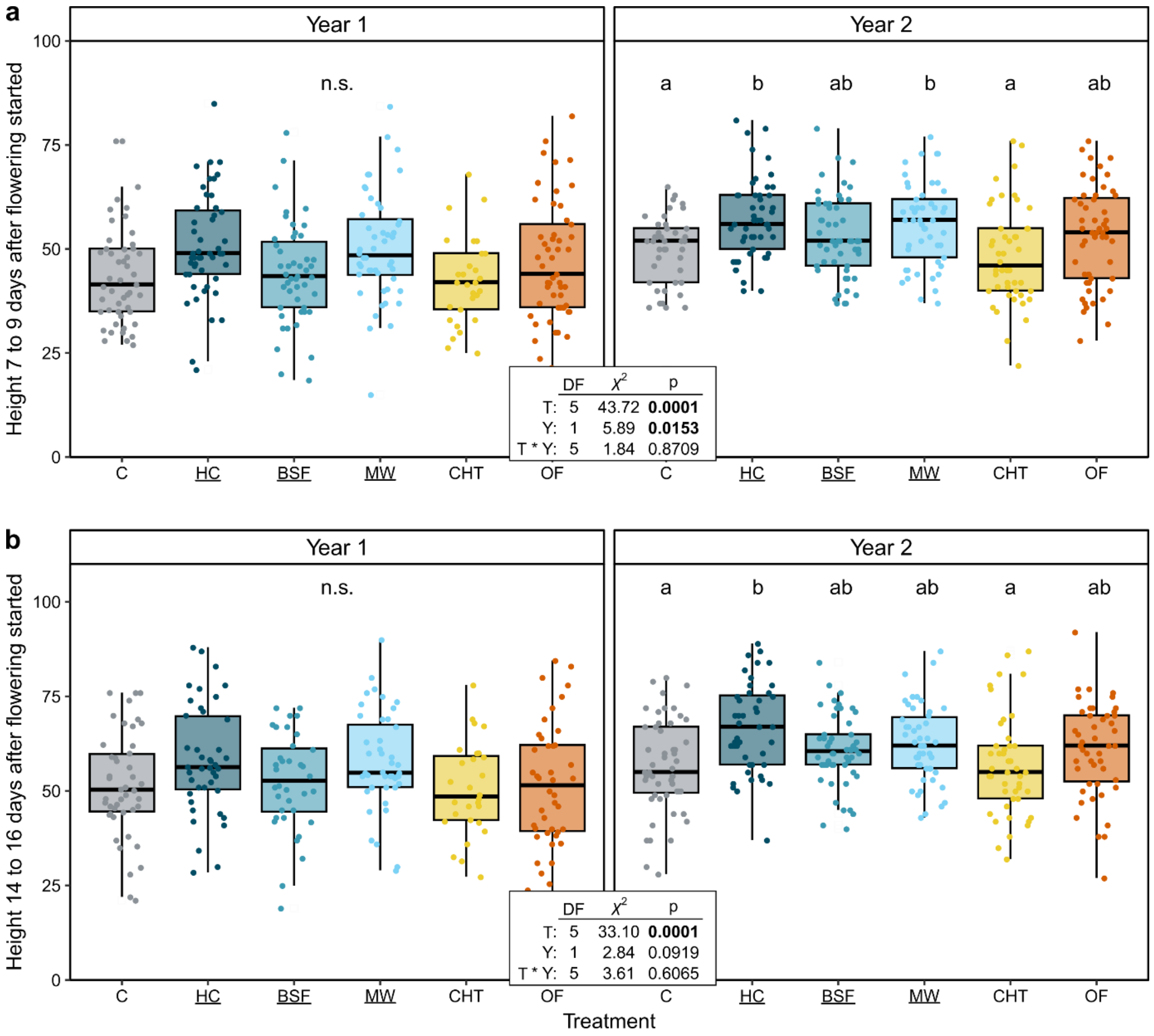

Supplementary Fig. 1 | Height of *Brassica nigra* plants grown in soil with different amendments in two years of field experiments. Plant height was measured (**a**) 7 to 9 days after flowering started and (**b**) 14 to 16 days after flowering started. Plants were grown in soil supplemented with exuviae of one of three insect species (underlined treatments), namely house cricket (HC), black soldier fly (BSF) or mealworm (MW). These are additionally compared to observations for plants grown in soil supplemented with either shrimp chitin (CHT), organic fertilizer (OF), or control plants (C) grown in untreated soil. Boxplots represent the median and interquartile range (IQR); whiskers represent 1.5 × IQR. Points represent individual observations. Results of a Generalized Least Square model are presented in the boxes, where T is main factor Treatment; Y is main factor Year, and T*Y is the interaction between Treatment and Year. Lower case letters above box plots indicate significant pairwise differences at $p < 0.05$ if no letters are shared, whereas n.s. indicates that no significant pair-wise differences were found. Sample sizes are provided in Supplementary Table 1

Supplementary Table 1 | Overview of sample sizes for each of the presented analyses. Each analysis is annotated with the experiment in which data was collected, *i.e.* Field experiment year 1, Field experiment year 2, data from field experiments aggregated for the two years, or data collected in the greenhouse experiment. For data collected in the field experiments, we also indicate whether data originated from observations made in early-season, late-season, at the end of the growing season, or accounts for all data collected throughout the season (global). Soil treatments are abbreviated as control (C), house cricket exuviae (HC), black soldier fly exuviae (BSF), mealworm exuviae (MW), shrimp chitin (CHT), and organic fertilizer (OF). Pollinator metrics refer to both the analysis of the total number of pollinators and the number of flowers visited per tracked pollinator. Microbial community refers to all analyses related to the microbial community structure and diversity, including Fisher and Shannon diversity and network analyses.

			**Treatment**					
**Analysis**	**Experiment**	**Sample time**	**C**	**HC**	**BSF**	**MW**	**CHT**	**OF**
Plant height	Field year 1	Early season	47	48	47	48	29	53
		Late season	46	42	36	44	28	42
	Field year 2	Early season	43	50	53	49	46	52
		Late season	51	44	50	47	44	47
Plant width	Field year 1	Early season	63	64	63	64	41	62
		Late season	57	55	53	57	36	52
	Field year 2	Early season	65	67	64	62	65	64
		Late season	62	59	57	60	59	60
Number of flowers	Field year 1	Early season	47	48	47	48	29	53
		Late season	46	42	36	44	28	42
	Field year 2	Early season	43	50	53	49	46	52
		Late season	51	44	50	47	44	47
Days until flowering	Field year 1	Early season	55	62	60	63	39	58
	Field year 2	Early season	64	67	64	62	65	62
Plant multivariate phenotype	Field year 1	Early season	43	47	46	48	28	51
		Late season	45	42	35	44	27	42
	Field year 2	Early season	43	50	53	49	46	52
		Late season	51	44	48	47	42	46
Seed production	Field year 1	End season	39	36	38	39	23	45
	Field year 2	End season	36	42	35	45	35	43
Pollinators metrics	Field year 1	Aggregated	54	54	48	47	29	51
	Field year 2	Aggregated	60	58	61	57	58	61
Interaction network	Field year 1	Early season	41	44	43	41	25	41
		Late season	45	40	29	41	23	38
	Field year 2	Early season	43	50	53	49	46	52
		Late season	51	44	50	47	44	47
Path model	Field aggregated	Global	55	56	54	55	34	56
Microbial community	Greenhouse		8	7	7	6	7	5

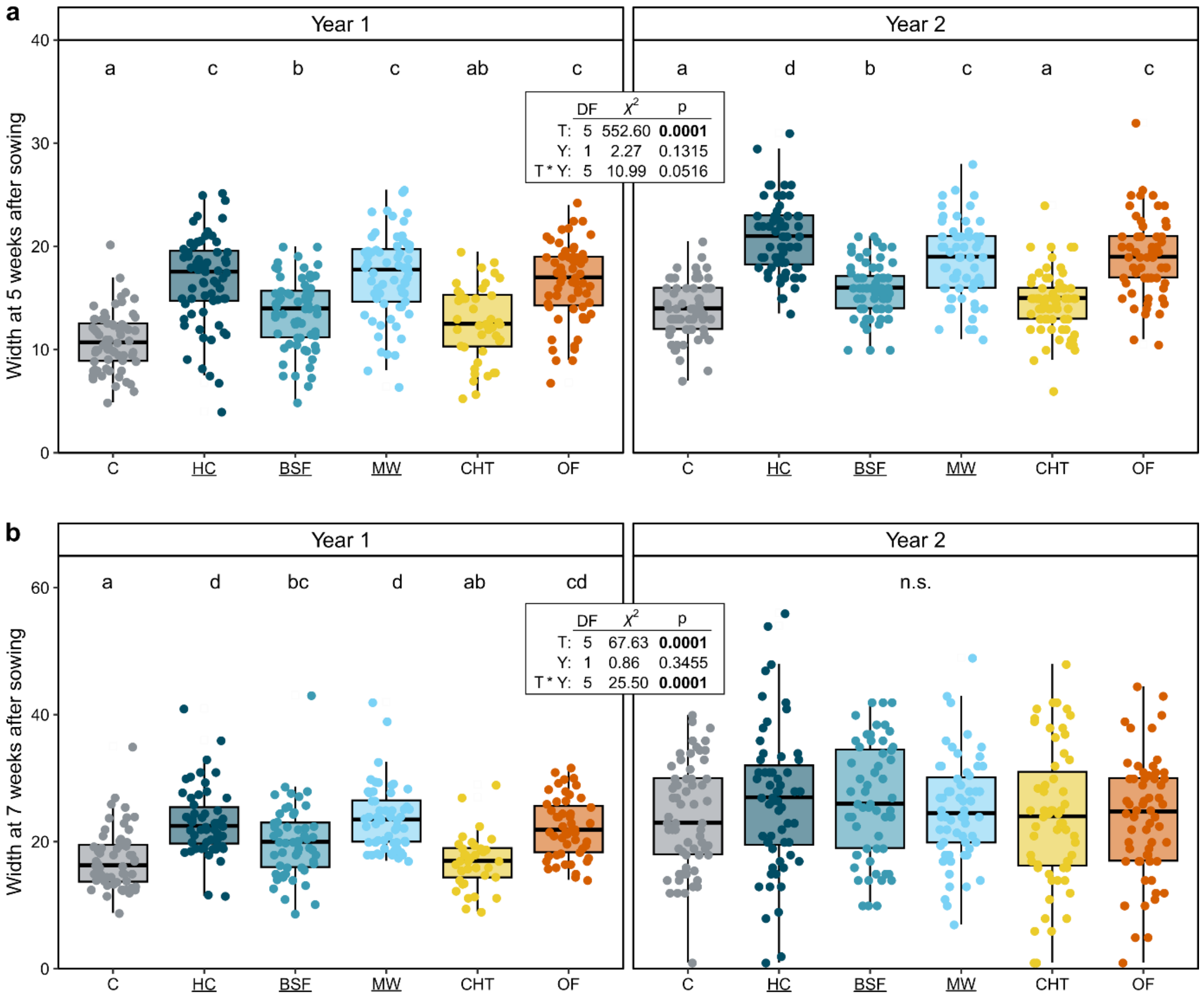

Supplementary Fig. 2 | Width of *Brassica nigra* plants grown in soil with different amendments in two years of field experiments. Plant width was measured (**a**) 5 weeks after sowing and (**b**) 7 weeks after sowing. Plants were grown in soil supplemented with exuviae of one of three different insect species (underlined treatments), namely house cricket (HC), black soldier fly (BSF) or mealworm (MW). These are compared to observations for plants grown in soil supplemented with either shrimp chitin (CHT), organic fertilizer (OF), or control plants (C) grown in untreated soil. Boxplots represent the median and interquartile range (IQR); whiskers represent 1.5 × IQR. Points represent individual observations. Results of a Generalized Least Square model are presented in the boxes, where T is main factor Treatment; Y is main factor Year, and T*Y is the interaction between Treatment and Year. Lower case letters above box plots indicate significant pairwise differences at $p < 0.05$ if no letters are shared, whereas n.s. indicates that no significant pair-wise differences were found. Sample sizes are provided in Supplementary Table 1

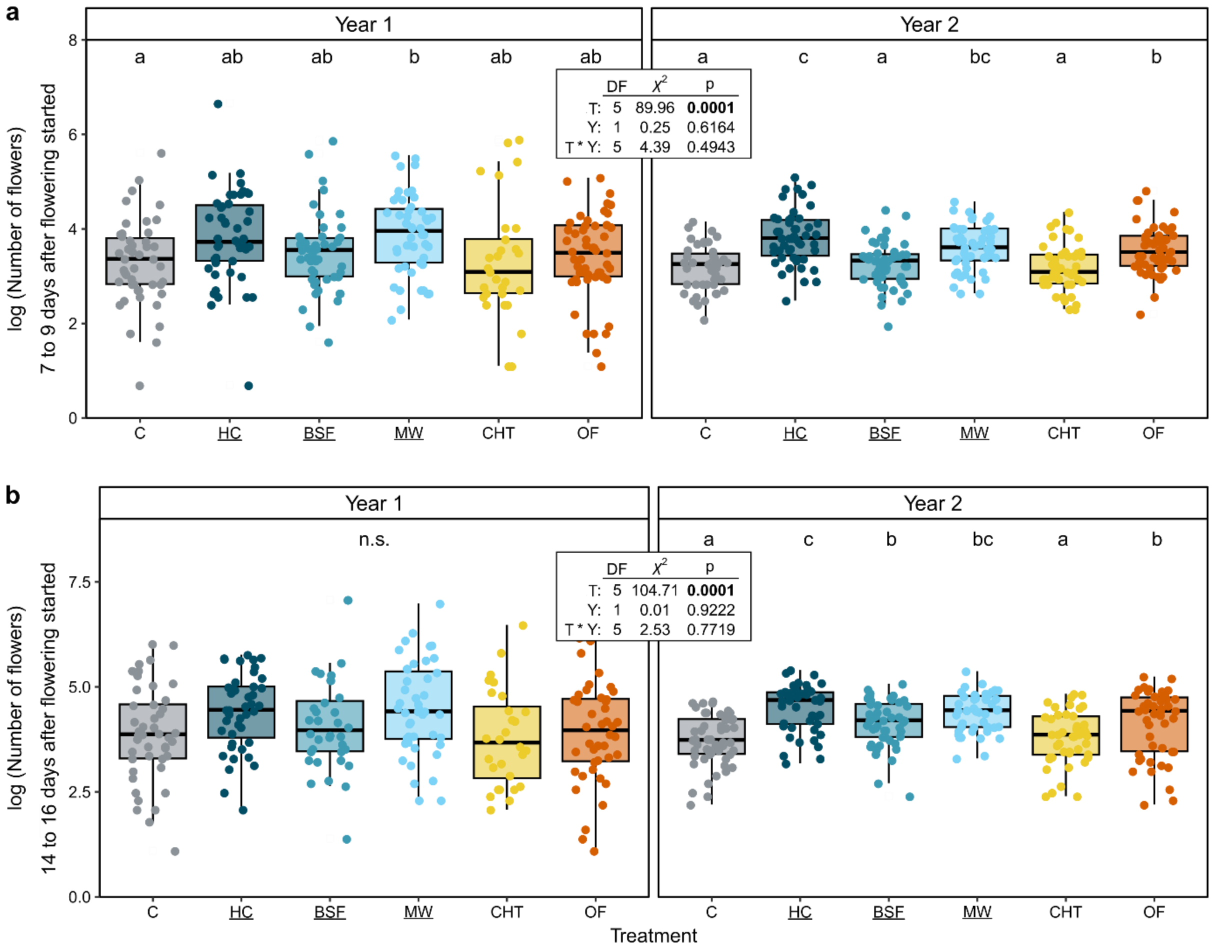

Supplementary Fig. 3 | Number of flowers produced by *Brassica nigra* plants grown in soil with different amendments in two years of field experiments. The number of flowers was counted (**a**) 7 to 9 days after flowering started and (**b**) 14 to 16 days after flowering started. Plants were grown in soil supplemented with exuviae of one of three different insect species (underlined treatments), namely house cricket (HC), black soldier fly (BSF) or mealworm (MW). These are compared to observations for plants grown in soil supplemented with either shrimp chitin (CHT), organic fertilizer (OF), or control plants (C) grown in untreated soil. Boxplots represent the median and interquartile range (IQR); whiskers represent 1.5 × IQR. Points represent individual observations. Results of a Generalized Least Square model are presented in the boxes, where T is main factor Treatment; Y is main factor Year, and T*Y is the interaction between Treatment and Year. Lower case letters above box plots indicate significant pairwise differences at $p < 0.05$ if no letters are shared, whereas n.s. indicates that no significant pair-wise differences were found. Sample sizes are provided in Supplementary Table 1

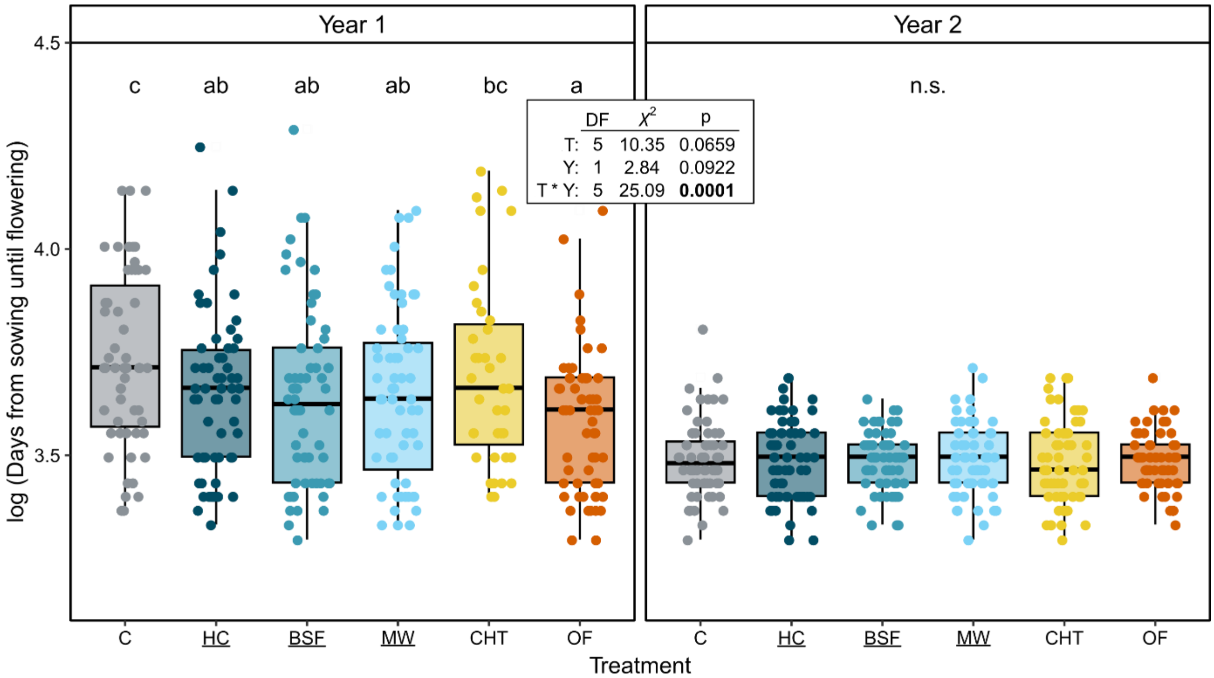

Supplementary Fig. 4 | Number of days from sowing until flowering started for *Brassica nigra* plants grown in soil with different amendments in two years of field experiments. Plants were grown in soil supplemented with exuviae of one of three different insect species (underlined treatments), namely house cricket (HC), black soldier fly (BSF) or mealworm (MW). These are compared to observations for plants grown in soil supplemented with either shrimp chitin (CHT), organic fertilizer (OF), or control plants (C) grown in untreated soil. Boxplots represent the median and interquartile range (IQR); whiskers represent 1.5 × IQR. Points represent individual observations. Results of a Generalized Least Square model are presented in the boxes, where T is main factor Treatment; Y is main factor Year, and T*Y is the interaction between Treatment and Year. Lower case letters above box plots indicate significant pairwise differences at $p < 0.05$ if no letters are shared, whereas n.s. indicates that no significant pair-wise differences were found. Sample sizes are provided in Supplementary Table 1

Supplementary Table 2 | Pairwise comparisons of the multivariate phenotypes of *Brassica nigra* plants grown in soil with different amendments in the field. Plants were grown in soil supplemented with exuviae of one of three different insect species (underlined treatments), namely house cricket (HC), black soldier fly (BSF) or mealworm (MW), were grown in soil supplemented with either shrimp chitin (CHT) or organic fertilizer (OF), or were grown in untreated soil as control (C). Pairwise comparisons were performed separately for measurements taken (**a**) early in the development of plants in the first year of field experiments, (**b**) early in the development of plants in the second year of field experiments, (**c**) late in the development of plants in the first year of field experiments, and (**d**) late in the development of plants in the second year of field experiments. Differences among treatments were estimated using a PERMANOVA. Values above the diagonal represent the p-values for each comparison, adjusted for multiple testing with false discovery rate corrections. Significant p values ($p < 0.05$) are indicated in bold. Values below the diagonal represent the respective pseudo-*F* values.

a

	C	HC	BSF	MW	CHT	OF
C		**0.006**	0.245	**0.004**	0.762	**0.004**
HC	5.61		**0.004**	0.762	**0.015**	0.245
BSF	1.15	5.76		**0.004**	0.523	0.245
MW	12.85	0.42	5.41		**0.010**	0.651
CHT	0.28	4.45	0.63	2.48		**0.024**
OF	7.38	1.30	1.48	0.53	3.45	

b

	C	HC	BSF	MW	CHT	OF
C		**0.002**	0.181	**0.002**	0.758	**0.002**
HC	15.49		**0.002**	0.415	**0.002**	0.280
BSF	1.73	8.95		**0.002**	0.181	**0.003**
MW	9.06	0.88	5.23		**0.002**	0.758
CHT	0.38	13.99	1.52	7.35		**0.002**
OF	8.51	1.28	4.52	0.35	6.52	

c

	C	HC	BSF	MW	CHT	OF
C		**0.007**	0.266	**0.007**	0.537	**0.033**
HC	9.03		0.160	0.421	0.132	0.160
BSF	1.38	2.10		0.450	0.160	0.450
MW	8.04	1.02	0.95		**0.010**	**0.033**
CHT	0.90	6.58	2.49	11.07		0.160
OF	3.80	2.26	0.74	4.85	1.85	

d

	C	HC	BSF	MW	CHT	OF
C		**0.005**	**0.019**	**0.005**	0.468	**0.047**
HC	7.48		**0.047**	0.252	**0.005**	0.054
BSF	4.65	2.68		0.055	0.262	0.468
MW	7.58	1.14	1.93		0.096	0.597
CHT	0.74	6.31	1.00	1.91		0.054
OF	2.92	2.36	0.69	0.34	2.73	

Supplementary Table 3 | Number of pollinators observed visiting flowers of *Brassica nigra* plants in two years of field experiments. Observations were made 7 to 9 days after flowering started and 14 to 16 days after flowering started. On average, flowering started 4 weeks and 4 days after sowing. Plants were grown in soil supplemented with exuviae of one of three insect species (underlined treatments), namely house cricket (HC), black soldier fly (BSF) or mealworm (MW), were grown in soil supplemented with either shrimp chitin (CHT) or organic fertilizer (OF) or were grown in untreated soil as control (C). Pollinators were classified in one of five groups, namely syrphid flies, other flies, honeybees, solitary bees, and bumblebees. We observed a total of 4011 pollinators over the two years of field experiments, of which 1660 were classified as syrphid flies, 690 as other flies, 1361 as honeybees, 207 as solitary bees, and 93 as bumblebees.

			Treatment					
**Year**	**Time after flowering started**	**Pollinator group**	C	HC	BSF	MW	CHT	OF
Year 1	7 to 9 days	Syrphid flies	79	99	90	65	61	66
		Other flies	14	29	28	19	15	31
		Honeybees	61	110	67	102	49	50
		Solitary bees	8	13	13	10	10	8
		Bumblebees	7	7	9	5	5	7
	14 to 16 days	Syrphid flies	103	111	117	173	38	98
		Other flies	15	48	10	31	6	28
		Honeybees	121	82	49	81	47	82
		Solitary bees	4	13	15	27	5	10
		Bumblebees	16	8	5	9	2	6
Year 2	7 to 9 days	Syrphid flies	103	111	117	173	38	98
		Other flies	15	48	10	31	6	28
		Honeybees	121	82	49	81	47	82
		Solitary bees	4	13	15	27	5	10
		Bumblebees	16	8	5	9	2	6
	14 to 16 days	Syrphid flies	59	52	33	45	33	48
		Other flies	41	47	57	60	36	37
		Honeybees	27	59	48	36	39	31
		Solitary bees	4	6	4	11	6	7
		Bumblebees	0	3	0	1	1	1

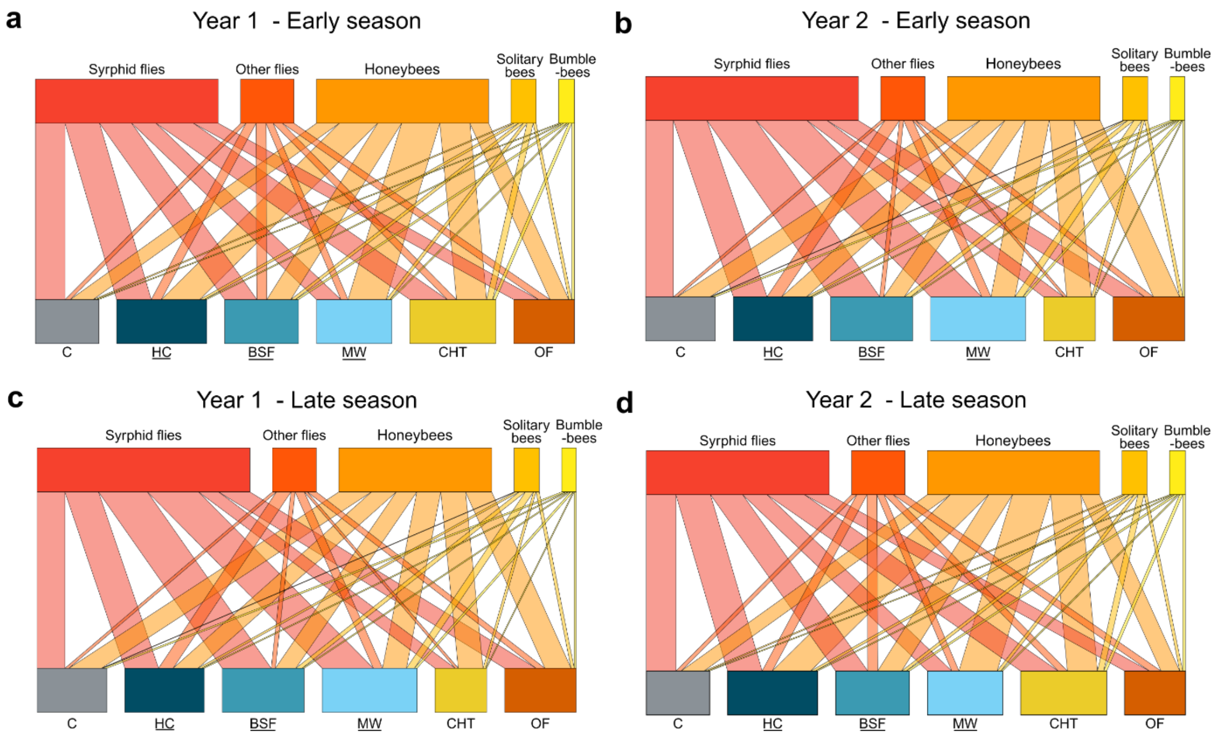

Supplementary Fig. 5 | Visualization of interaction networks between *Brassica nigra* plants grown in soil with different amendments and the observed pollinator community in two years of field experiments. The different panels represent observations taken at (**a**) 7 to 9 days after individual plants started flowering in the first year of field experiments, (**b**) 7 to 9 days after flowering started in the second year of field experiments, (**c**) 14 to 16 days after plants started flowering in the first year of field experiments, and at (**d**) 14 to 16 days after flowering started in the second year of field experiments. On average, flowering started at 4 weeks and 4 days after sowing. Pollinators were classified in one of five groups, namely syrphid flies, other flies, honeybees, solitary bees, and bumblebees. Plants were grown in soil supplemented with exuviae of one of three different insect species (underlined treatments), namely house cricket (HC), black soldier fly (BSF) or mealworm (MW), were grown in soil supplemented with either shrimp chitin (CHT), or organic fertilizer (OF), or were grown in untreated soil as control (C). The bipartite interaction networks are constructed by dividing the summed number of observed interactions with plants in each of the soil treatments for each pollinator group (upper boxes in the interaction networks, Supplementary Table 2) by the number of monitored plants in each of the soil treatments (lower boxes in the interaction networks). Networks thus reflect the average community of pollinators interacting with an individual plant growing in each of the differently treated soils. Connections between the upper and lower boxes represent interactions, with the width of these shaded connections scaled to represent the average number of pollinators belonging to the pollinator group interacting with an individual plant of each treatment. Boxes representing the five different pollinator groups and boxes representing the plants growing soil with different amendments are scaled by the number of interactions they were part of. Sample sizes are provided in Supplementary Table 1

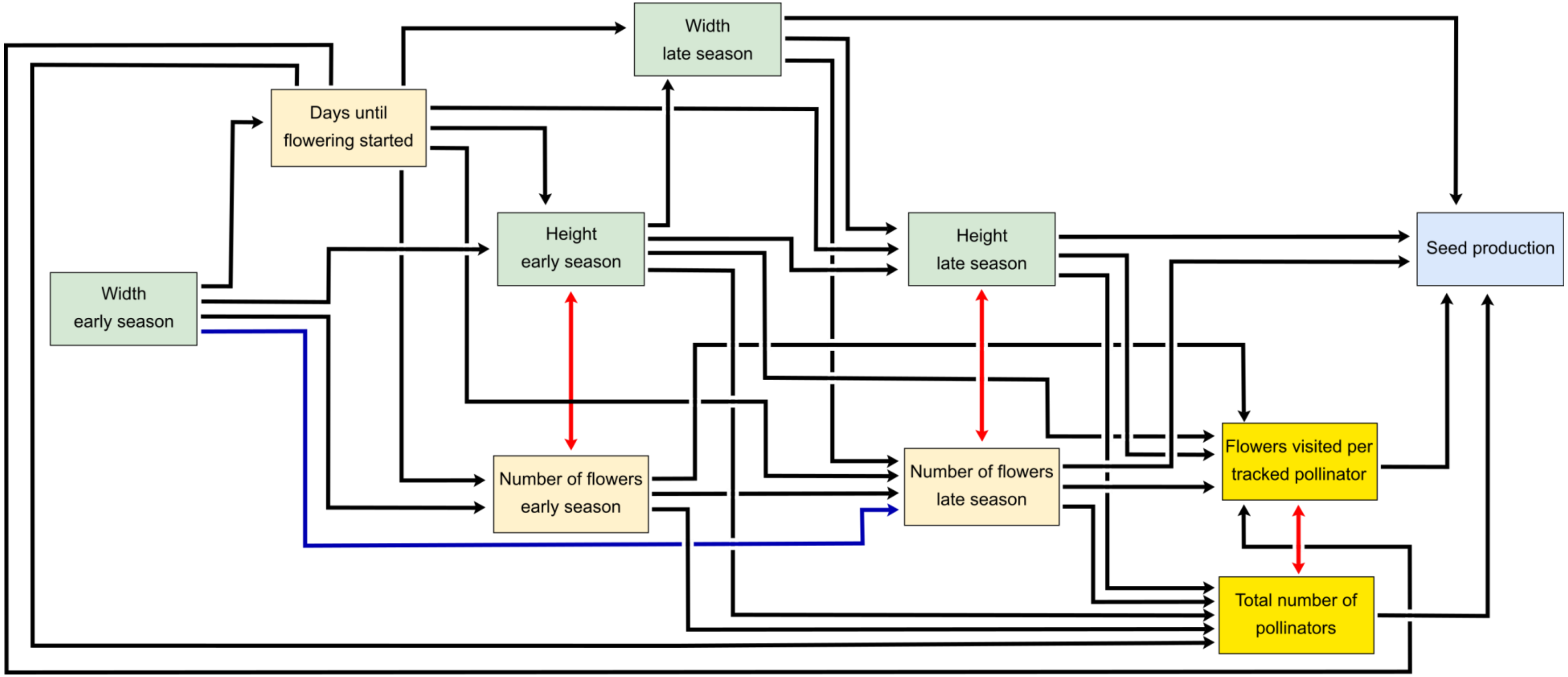

Supplementary Fig. 6 | Path diagram of the causal model expressing the relation between the measured proxies for plant growth and development, the associated pollinator community, and seed production of *Brassica nigra* plants in the field experiment. Plant width was measured at 5 and at 7 weeks after sowing (early and late, respectively), and the number of flowers were counted, and the height was measured between 7 to 9 days and between 14 to 16 days after flowering started (early and late, respectively). The total number of pollinators and the number of flowers visited per tracked pollinator are calculated from observations which were aggregated for the two observation rounds (between 7 to 9 days and between 14 to 16 days after flowering started). On average, flowering started 4 weeks and 4 days after sowing. Variables related to plant biomass (i.e. plant height and width), flowering, the associated pollinator community, and seed production are represented in green, orange, yellow, and blue rectangles, respectively. Black arrows represent unidirectional causal relationships among variables. Double-headed red arrows show relations for which we could not presume a causal effect, and which were included as dependent errors. The blue arrow represents a relation which was not included in our initial causal hypothesis, but which proved a necessary addition to the model to ensure data were sufficiently well represented by the path model.

Supplementary Table 4 | Overview of the fit of the path model to data obtained for *Brassica nigra* plants growing in soil with different amendments in the field experiment. Plants were grown in soil supplemented with exuviae of one of three different insect species (underlined treatments), namely house cricket (HC), black soldier fly (BSF) or mealworm (MW), were grown in soil supplemented with either shrimp chitin (CHT) or organic fertilizer (OF), or were grown in untreated soil as control (C). How well the path model describes the observed data is expressed by Fisher's C global goodness of fit statistic with the associated degrees of freedom and p-value. A p-value > 0.05 allows us to accept the hypothesis that the observed data were generated by the causal mechanism presented in Supplemental Figure 8, indicating that the data are sufficiently well represented by the path model.

**Treatment**	**Fisher's C**	**DF**	**p**
C	24.44	24	0.437
HC	23.99	24	0.462
BSF	23.46	24	0.493
MW	21.16	24	0.629
CHT	33.75	24	0.089
OF	30.38	24	0.173

Supplementary Table 5 | Overview of the standardized path coefficients estimating the sign and strength of the causal relations between the measured proxies for plant growth and development, the associated pollinator community, and seed production of *Brassica nigra* plants in the field experiment. Plants were grown in soil supplemented with exuviae of one of three different insect species (underlined treatments), namely house cricket (HC), black soldier fly (BSF) or mealworm (MW), were grown in soil supplemented with either shrimp chitin (CHT) or organic fertilizer (OF), or were grown in untreated soil as control (C). Plant width was measured at 5 and at 7 weeks after sowing (early and late, respectively), while the number of flowers were counted and the height was measured between 7 to 9 days and between 14 to 16 days after flowering started (early and late, respectively). On average, flowering started 4 weeks and 4 days after sowing. The total number of pollinators and the number of flowers visited per tracked pollinator are calculated from observations which were aggregated for the two observation rounds.

		Treatment					
**Predictor**	**Response**	C	HC	BSF	MW	CHT	OF
Width early season	Days until flowering started	0.08	0.24	0.00	-0.01	0.08	-0.01
Width early season	Number of flowers early season	0.14	0.29	0.08	-0.26	0.13	0.10
Days until flowering started	Number of flowers early season	0.35	0.37	0.19	-0.01	0.53	0.16
Days until flowering started	Number of flowers late season	0.37	0.12	0.08	0.17	0.31	0.14
Width early season	Number of flowers late season	0.06	0.02	0.167	0.23	0.09	-0.12
Width late season	Number of flowers late season	0.30	0.24	0.12	0.22	0.02	0.36
Number of flowers early season	Number of flowers late season	0.21	0.42	0.29	0.39	0.34	0.34
Height early season	Width late season	-0.02	0.01	0.10	0.28	0.10	0.27
Days until flowering started	Width late season	0.10	0.31	0.35	0.19	0.09	0.21
Width early season	Width late season	0.22	0.33	0.25	0.20	0.41	0.30
Days until flowering started	Height early season	0.39	0.24	0.27	0.27	0.72	0.52
Width early season	Height early season	0.20	0.26	0.37	0.47	0.39	0.44
Days until flowering started	Height late season	0.24	-0.01	-0.22	0.05	0.08	0.08
Width late season	Height late season	0.14	0.02	0.06	0.14	0.24	0.16
Height early season	Height late season	0.63	0.85	0.79	0.77	0.81	0.75
Days until flowering started	Total number of pollinators	0.28	0.16	-0.04	0.25	0.34	0.13
Number of flowers early season	Total number of pollinators	0.02	0.14	0.11	0.12	-0.05	0.31
Number of flowers late season	Total number of pollinators	0.35	0.16	0.15	0.13	0.10	0.23
Height early season	Total number of pollinators	0.06	0.13	-0.04	-0.02	-0.06	-0.18
Height late season	Total number of pollinators	-0.11	-0.08	0.06	-0.03	0.08	0.26
Days until flowering started	Flowers visited per tracked pollinator	0.47	0.20	0.19	0.19	-0.38	0.12
Number of flowers early season	Flowers visited per tracked pollinator	-0.39	0.12	0.20	0.20	0.62	0.05
Number of flowers late season	Flowers visited per tracked pollinator	0.38	0.36	-0.25	0.05	-0.18	0.40
Height early season	Flowers visited per tracked pollinator	0.33	-0.34	-0.46	0.13	0.24	0.20
Height late season	Flowers visited per tracked pollinator	0.04	0.17	0.40	-0.16	-0.11	-0.30
Number of flowers late season	Seed production	0.46	0.18	0.68	0.26	0.09	0.08
Height late season	Seed production	-0.07	-0.02	-0.03	-0.02	0.04	0.04
Width late season	Seed production	0.29	0.02	-0.05	0.28	0.07	0.39
Flowers visited per tracked pollinator	Seed production	-0.25	0.02	0.04	0.22	-0.07	0.01
Total number of pollinators	Seed production	-0.40	0.23	-0.12	0.19	0.07	-0.03

Supplementary Table 6 | Pairwise comparison of the structure of microbial communities in the rhizosphere of *Brassica nigra* plants grown in soil with different amendments in the greenhouse. Plants were grown in soil supplemented with exuviae of one of three different insect species (underlined treatments), namely house cricket (HC), black soldier fly (BSF) or mealworm (MW), were grown in soil supplemented with either shrimp chitin (CHT) or organic fertilizer (OF), or were grown in untreated soil as control (C). Differences among treatments were estimated using a PERMANOVA, with p-values for each comparison adjusted for multiple testing with false discovery rate corrections.

Comparison			Sum of squares	Mean sum of squares	*Pseudo*- F	R^2	p	Adjusted p
BSF	<->	C	0.50	0.50	4.97	0.28	0.0020	0.0033
BSF	<->	CHT	0.29	0.29	3.29	0.22	0.0020	0.0033
BSF	<->	HC	0.33	0.33	4.76	0.28	0.0020	0.0033
BSF	<->	MW	0.20	0.20	2.65	0.19	0.0020	0.0033
BSF	<->	OF	0.66	0.66	6.91	0.41	0.0030	0.0041
C	<->	CHT	0.23	0.23	2.01	0.13	0.0120	0.0120
C	<->	HC	0.34	0.34	3.63	0.22	0.0010	0.0033
C	<->	MW	0.28	0.28	2.85	0.19	0.0040	0.0043
C	<->	OF	0.39	0.39	3.17	0.22	0.0040	0.0043
CHT	<->	HC	0.26	0.26	3.10	0.21	0.0010	0.0033
CHT	<->	MW	0.19	0.19	2.15	0.16	0.0040	0.0043
CHT	<->	OF	0.41	0.41	3.75	0.27	0.0020	0.0033
HC	<->	MW	0.16	0.16	2.39	0.18	0.0010	0.0033
HC	<->	OF	0.31	0.31	3.59	0.26	0.0020	0.0033
MW	<->	OF	0.38	0.38	4.04	0.31	0.0030	0.0041

Supplementary Table 7 | Pairwise comparison of beta dispersion of rhizobial communities associated with the different soil treatments. Plants were grown in soil supplemented with exuviae of one of three different insect species (underlined treatments), namely house cricket (HC), black soldier fly (BSF) or mealworm (MW), were grown in soil supplemented with either shrimp chitin (CHT) or organic fertilizer (OF), or were grown in untreated soil as control (C). Values represent p-values of each pairwise comparison, adjusted for multiple testing using false discovery rate corrections. Values in bold are considered significant ($p < 0.05$). Exuviae-based soil treatments are indicated as underlined treatments.

	HC	BSF	MW	CHT	OF
C	**0.015**	0.136	**0.032**	0.858	0.700
HC		0.938	1.000	0.227	0.543
BSF			0.976	0.747	0.955
MW				0.340	0.668
CHT					0.999

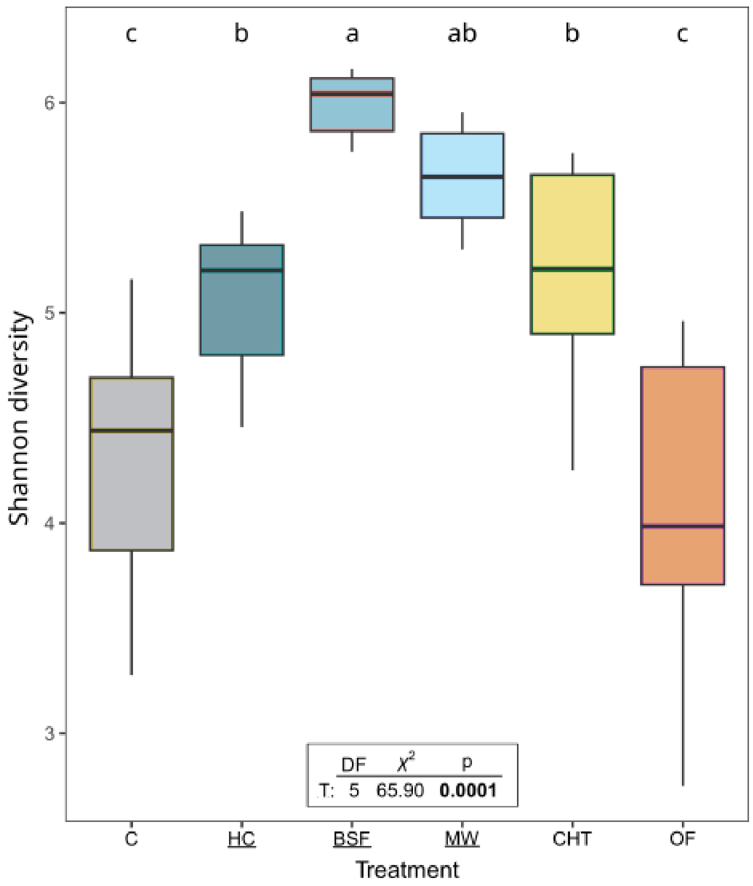

Supplementary Fig. 7 | Shannon's diversity index indicating the effect of different soil treatments on the diversity of soil microbial communities. Plants were grown in soil supplemented with exuviae of one of three different insect species (underlined treatments), namely house cricket (HC), black soldier fly (BSF) or mealworm (MW), were grown in soil supplemented with either shrimp chitin (CHT) or organic fertilizer (OF), or were grown in untreated soil as control (C). The box plots represent the median and interquartile range (IQR); whiskers represent 1.5 × IQR. The overall effect of soil supplementation on Shannon diversity index was assessed by a generalized least-square model. Lower case letters above box plots indicate significant pairwise differences at $p < 0.05$ if no letters are shared, using a Least Significant Difference test adjusted for multiple comparisons using false discovery rate.

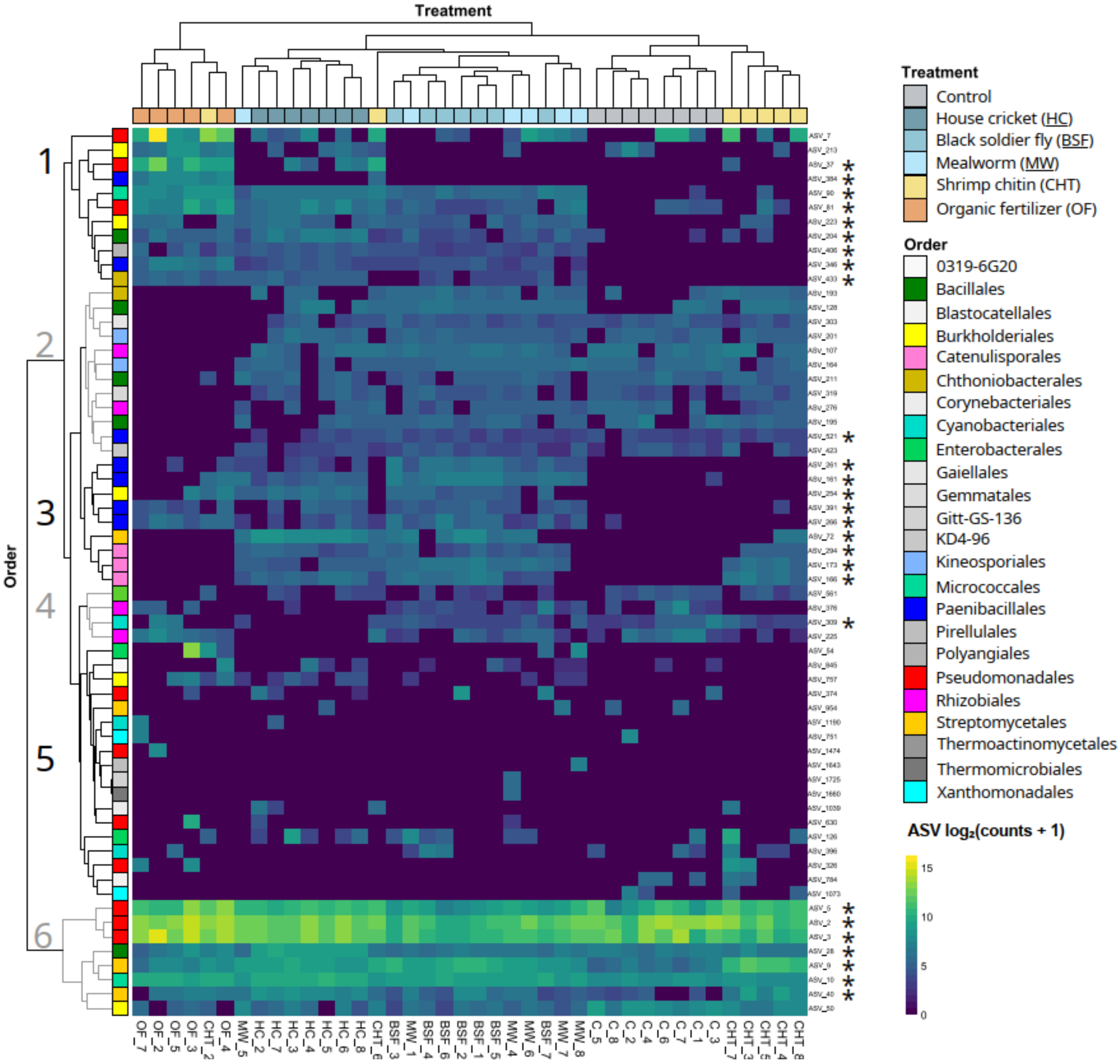

Supplementary Fig. 8 | Bi-cluster heatmap indicating the log_2-transformed abundance of 62 differentially abundant ASVs (rows) across the different *Brassica nigra* plants grown in soil with different amendments in the greenhouse. ASVs were identified using differential abundance testing. ASV clusters (complete linkage) are numbered and alternating in grey scale to facilitate identification. ASVs are further labeled by the order they belong to, with orders with a single ASV representative indicated in gray scale. Asterisks next to ASVs labels annotate ASVs that were also defined as important to separate treatments by the random forest model. Plants were grown in soil supplemented with exuviae of one of three different insect species (underlined treatments), namely house cricket (HC), black soldier fly (BSF) or mealworm (MW), or were grown in soil supplemented with either shrimp chitin (CHT) or organic fertilizer (OF), or were grown in untreated soil as control (C). Sample sizes are provided in Supplementary Table 1.

Supplementary Table 8 | ASVs identified as random forest predictors for both soil treatment and the number of flowers. The table presents the taxonomic classification and random forest model importance of the 13 ASVs identified as predictors for the two response variables (*i.e.* soil treatment and number of flowers). We indicated the genus name as NA when no reliable identification could be made. Importance indicates how much each ASV contributes to the model's predictive power in relation to soil treatment or the number of flowers plants produced.

**ASV**	**Domain**	**Phylum**	**Class**	**Order**	**Family**	**Genus**	**Mean importance for treatment**	**Mean importance for number of flowers**
**ASV_3**	Bacteria	Proteobacteria	Gammaproteobacteria	Pseudomonadales	Pseudomonadaceae	*Pseudomonas*	13.999	9.005
**ASV_10**	Bacteria	Actinobacteriota	Actinobacteria	Micrococcales	Intrasporangiaceae	*Terrabacter*	20.543	13.906
**ASV_26**	Bacteria	Actinobacteriota	Actinobacteria	Micrococcales	Intrasporangiaceae	*Phycicoccus*	9.798	8.259
**ASV_30**	Bacteria	Actinobacteriota	Actinobacteria	Propionibacteriales	Nocardioidaceae	*Nocardioides*	22.446	9.648
**ASV_36**	Bacteria	Firmicutes	Bacilli	Bacillales	Bacillaceae	*Bacillus*	22.620	20.943
**ASV_72**	Bacteria	Actinobacteriota	Actinobacteria	Streptomycetales	Streptomycetaceae	NA	13.633	13.856
**ASV_204**	Bacteria	Firmicutes	Bacilli	Bacillales	Planococcaceae	NA	19.869	21.862
**ASV_254**	Bacteria	Proteobacteria	Gammaproteobacteria	Burkholderiales	Oxalobacteraceae	*Massilia*	10.075	19.546
**ASV_266**	Bacteria	Firmicutes	Bacilli	Paenibacillales	Paenibacillaceae	*Paenibacillus*	21.457	13.942
**ASV_283**	Bacteria	Firmicutes	Bacilli	Alicyclobacillales	Alicyclobacillaceae	*Tumebacillus*	8.565	15.596
**ASV_346**	Bacteria	Firmicutes	Bacilli	Paenibacillales	Paenibacillaceae	*Paenibacillus*	15.606	20.875
**ASV_449**	Bacteria	Actinobacteriota	Actinobacteria	Corynebacteriales	Nocardiaceae	*Nocardia*	17.099	14.758
**ASV_511**	Bacteria	Actinobacteriota	Actinobacteria	Micromonosporales	Micromonosporacea e	NA	7.710	11.242

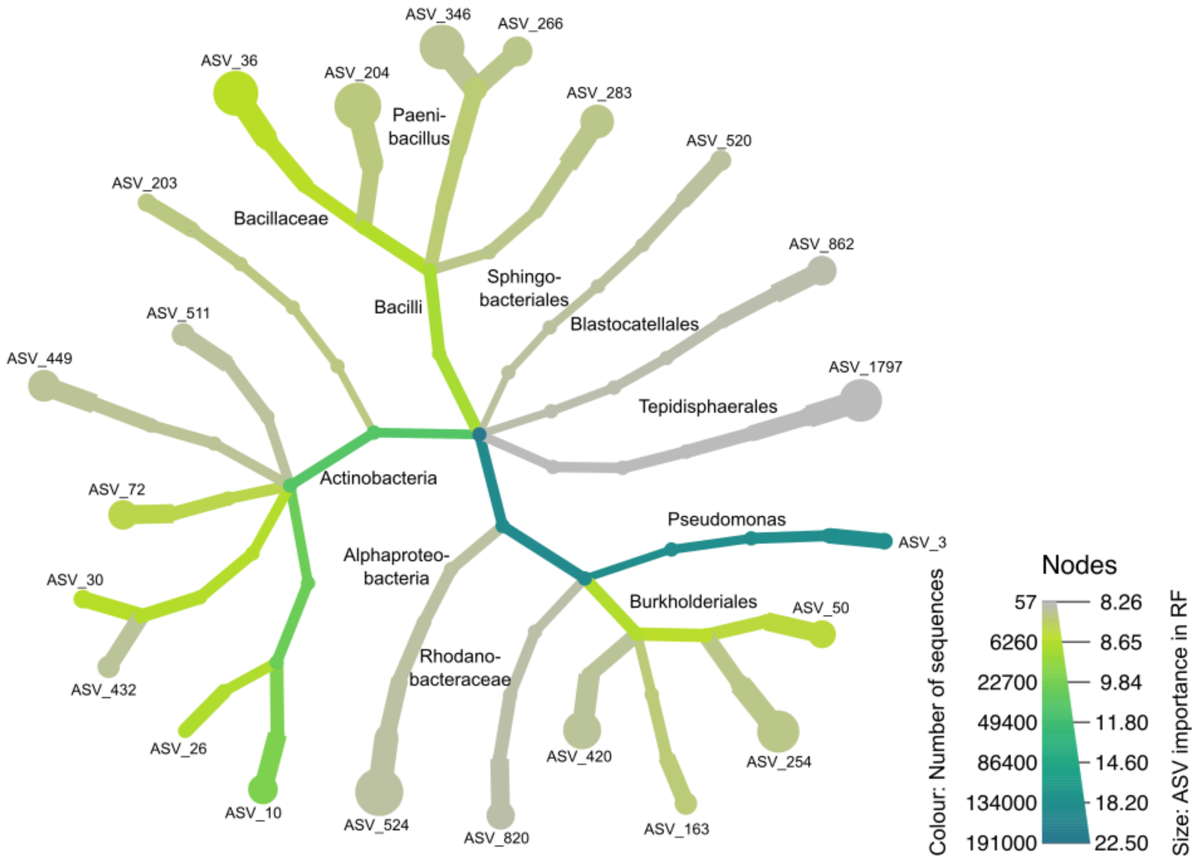

Supplementary Fig.9 | Heat tree showing the 23 ASVs that were important in predicting the number of flowers according to the random forest model. Each node represents a taxonomic group, from domain to ASV. Colors indicate log_2-transformed relative abundances, and size of the node represents the importance of the ASV in predicting the number of flowers according to the random forest model. A detailed figure is available in the online supplementary information. Sample sizes are provided in Supplementary Table 1.

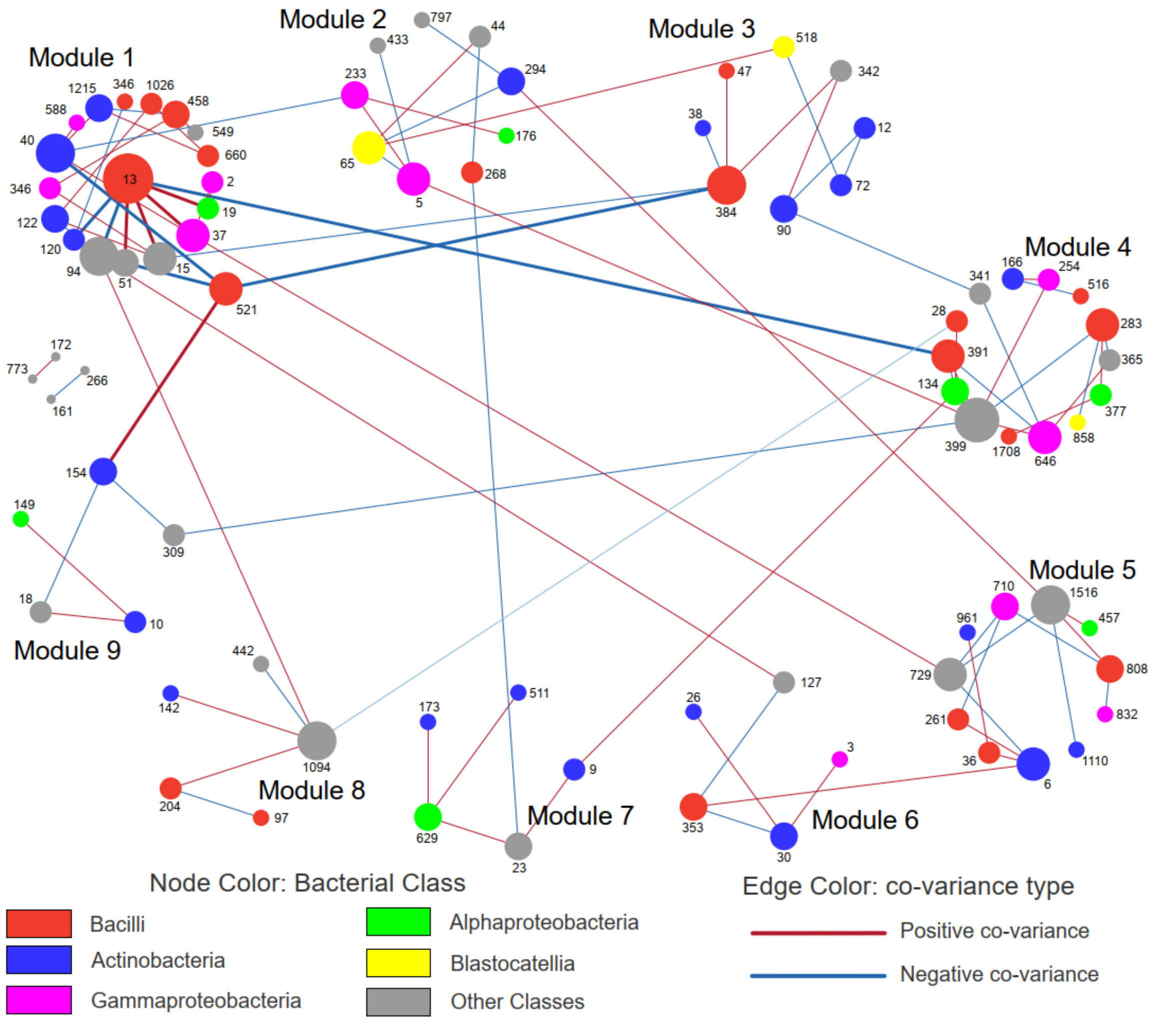

Supplementary Fig. 10 | Network showing co-occurrences of the 94 ASVs identified as important predictors of the soil treatment in the random forest analysis. Each dot (*i.e.* network node) represents an ASV (annotated with their respective number), with colors indicating microbial class and size of the dot indicating the number of connections (*i.e.* degree). Red lines indicate positive co-occurrences and blue lines indicate negative co-occurrences of ASVs in the microbial communities in the rhizosphere of *Brassica nigra* plants. Thicker lines represent connections with the module hub (ASV 13) or network connector (ASV 521). Network nodes are clustered in circles that form network modules (sub-community clusters of ASVs). Sample sizes are provided in Supplementary Table 1.

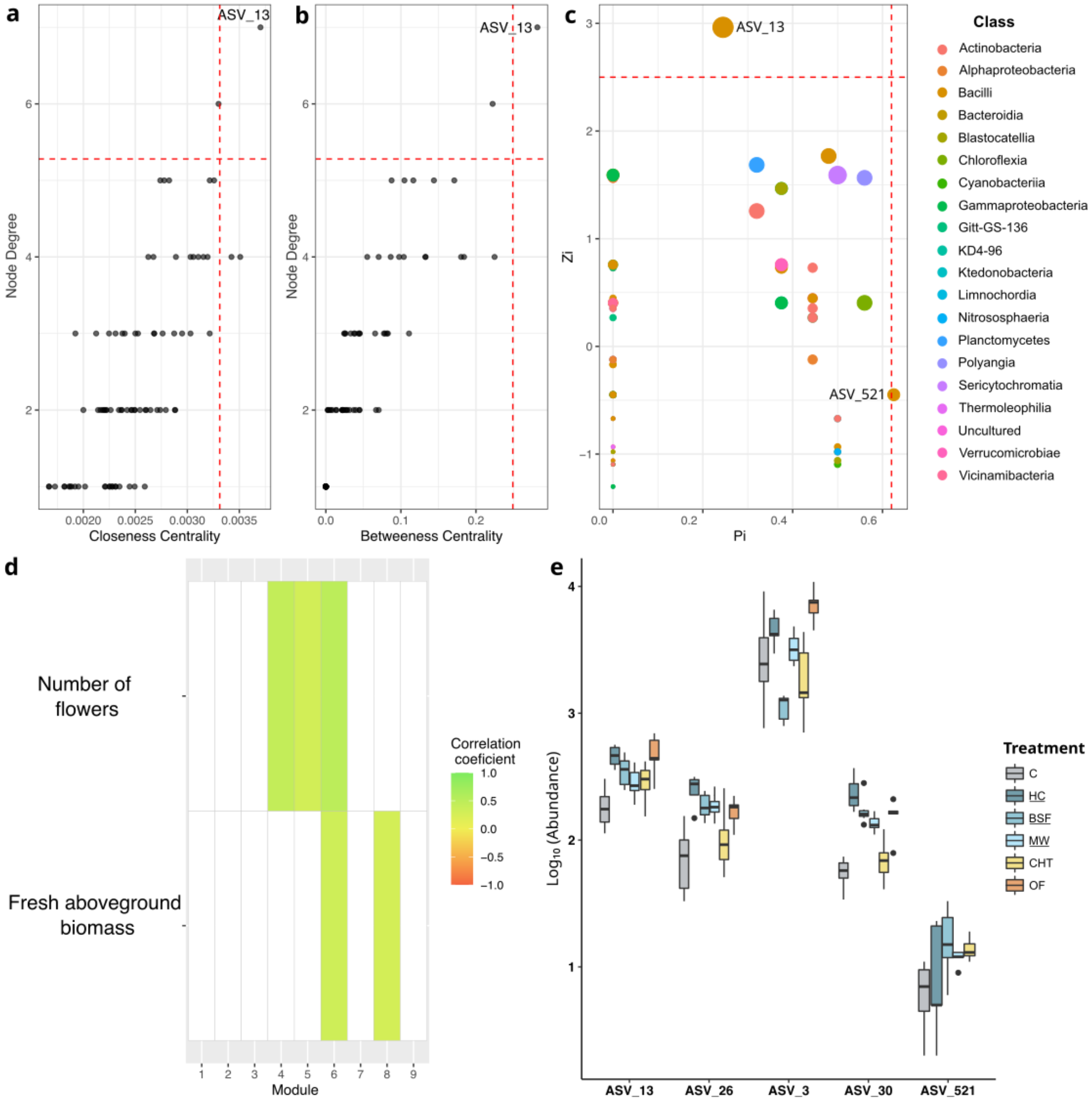

Supplementary Fig.11 | Overview of analysis of network metrics and relation to plant growth or development. (**a**) Keystone taxa are identified by a stronger than average correlation between node degree (*i.e.* the number of connections with other nodes) and closeness centrality (*i.e.* the shortest path length from the node to every other node in the network) and by (**b**) by node degree and betweenness centrality (*i.e.* the extent to which a certain node lies on the shortest paths between other nodes and thus connects nodes with each other). Additionally, to further identify nodes as module hubs or connectors, the (**c**) correlation between within-module connectivity (Zi) and between module connectivity (Pi) provides further support to the importance of specific nodes in the network. (**d**) Correlation between ASV composition of individual modules in the network and the observed plant phenotype using weighted correlation network analysis (WGCNA). (**e**) Log-transformed abundance of selected ASVs per treatment. Sample sizes are provided in Supplementary Table 1.

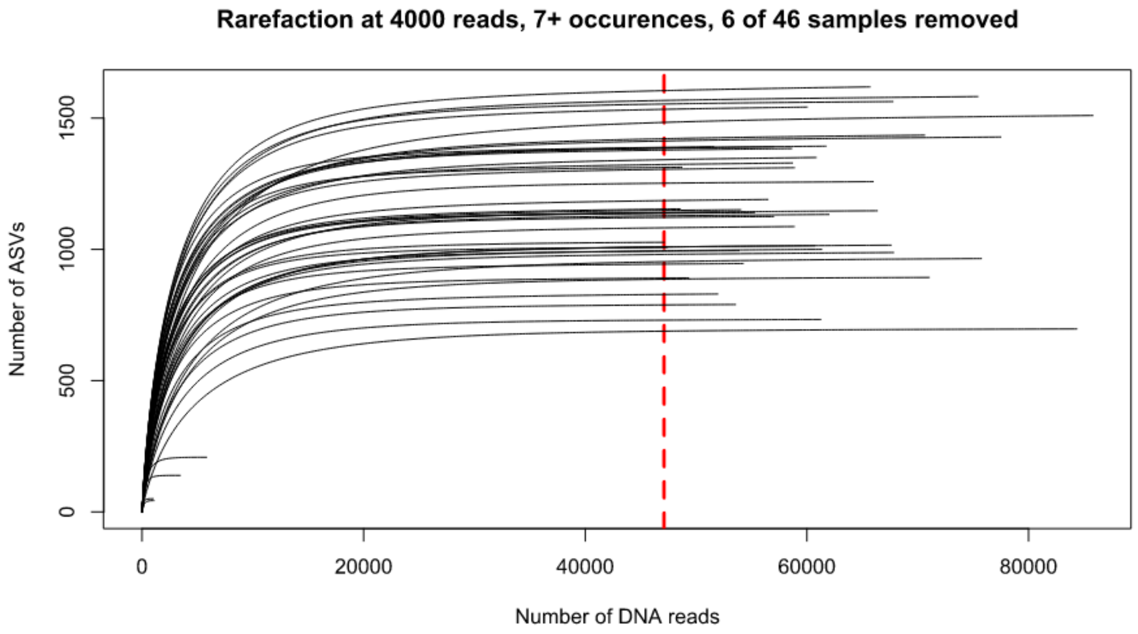

Supplementary Fig. 12 | Rarefaction curve showing library size and ASV diversity per sample (black lines). Six samples with less than 6.000 reads were removed from the analysis. Libraries were rarefied to at least 47.124 reads per sample (threshold indicated by the red dashed line).

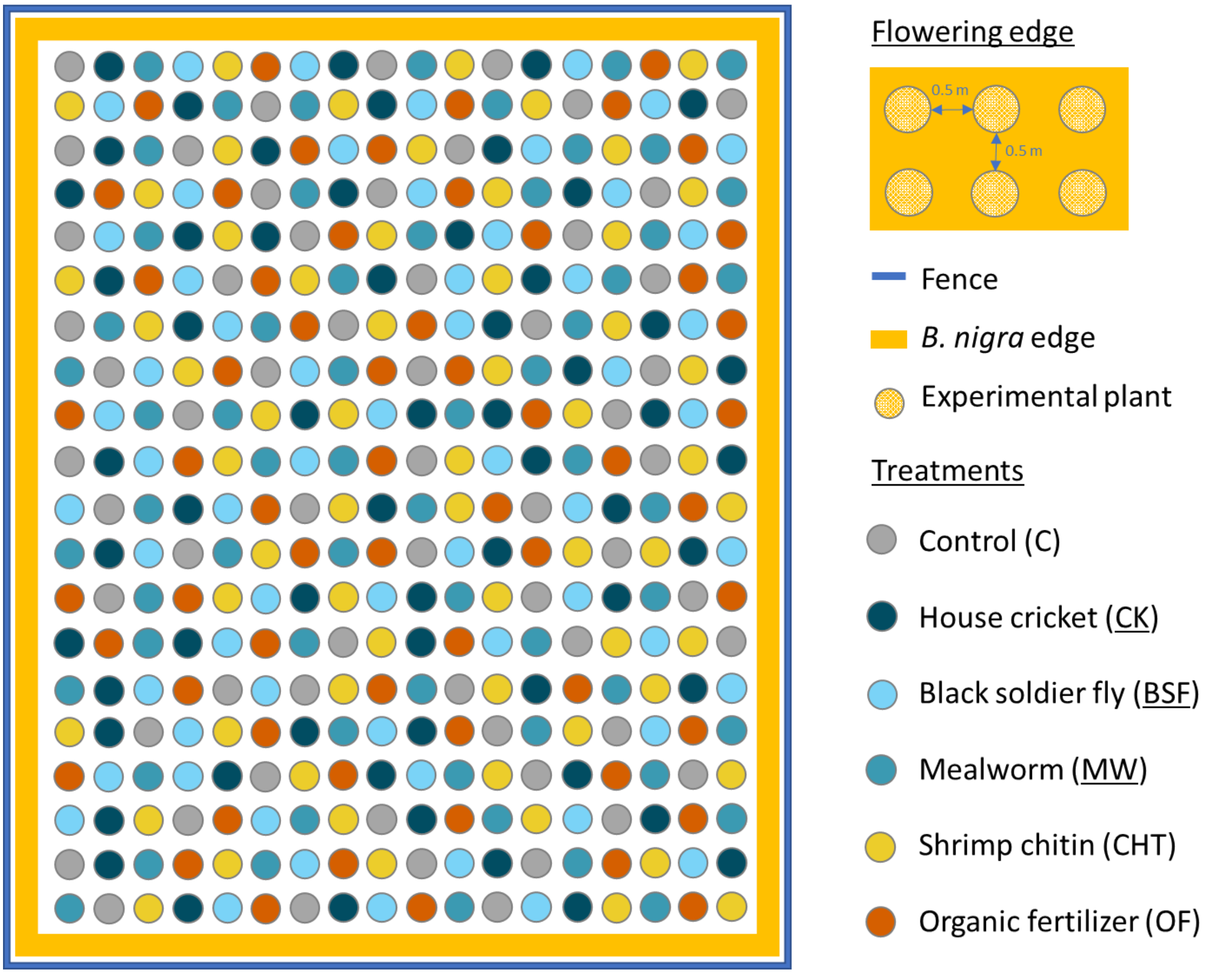

Supplementary Fig. 13 | Field design used in both years of field experiments. The 32 m x 20 m field was surrounded by a flowering edge (yellow border) of *Brassica nigra* and a meshed-wire fence (blue line). Experimental plants were randomly assigned to a treatment and planted in a randomized planting position in the field. Plants were grown in soil supplemented with exuviae of one of three different insect species (underlined treatments), were grown in soil supplemented with either shrimp chitin (CHT), organic fertilizer (OF), or grown in untreated soil as control (C).
